# Baseline profile peripheral Tfh cells predict immune-related adverse events in immune checkpoint inhibitor therapy of gastrointestinal cancer

**DOI:** 10.3389/fimmu.2025.1559275

**Published:** 2025-05-29

**Authors:** Yifan Wang, Zhening Zhang, Tong Xie, Yudong Liu, Cheng Zhang, Hao Li, Ruiling Feng, Bo Huang, Qinghong Liu, Naidi Wang, Xiaoyan Xing, Yipeng Han, Xue Li, Ruoyi Wang, Jing He, Zhi Peng

**Affiliations:** ^1^ Department of Rheumatology and Immunology, Peking University People’s Hospital, Beijing Key Laboratory for Rheumatism Mechanism and Immune Diagnosis, Beijing, China; ^2^ Key Laboratory of Carcinogenesis and Translational Research (Ministry of Education/Beijing), Department of Gastrointestinal Oncology, Peking University Cancer Hospital and Institute, Beijing, China; ^3^ The Key Laboratory of Geriatrics, Beijing Institute of Geriatrics, Beijing Hospital, National Center of Gerontology, National Health Commission, Institute of Geriatric Medicine, Chinese Academy of Medical Sciences, Beijing, China; ^4^ State Key Laboratory of Holistic Integrative Management of Gastrointestinal Cancers, Beijing Key Laboratory of Carcinogenesis and Translational Research, Department of Gastrointestinal Oncology, Peking University Cancer Hospital and Institute, Beijing, China

**Keywords:** immune checkpoint inhibitors, immune-related adverse events, gastrointestinal cancer, T cell subsets, Tfh cells, Tph cells, predictive models

## Abstract

**Background:**

Immune checkpoint inhibitors (ICIs) have transformed cancer therapy but are limited by immune-related adverse events (irAEs). This study aimed to assess peripheral T cell profiles to identify irAEs biomarkers and construct predictive models.

**Methods:**

In our study, we enrolled and followed 51 gastrointestinal cancer patients receiving anti-PD-1/PD-L1 therapies, with 22 developed irAEs (AE) and 29 didn’t (NAE). We examined their peripheral blood using Olink technology, RNA-seq, and flow cytometry to explore the immunological characteristics of their circulating environment before and after early stages of ICIs treatment.

**Results:**

Our study discovered after early stages of ICIs treatment, a stronger upregulation of T cell activation genes, particularly Tfh-associated genes, was observed in AE patients. Flow cytometry result confirmed that AE patients exhibited elevated CD4^+^CXCR5^-^ICOS^+^ cells (p<0.01), CD4^+^CXCR5^+^ICOS^+^ cells (p<0.05) and Th1/Th2 ratio (p<0.05) after early stages. At baseline, AE patients had higher levels of serum inflammatory proteins including IL-12β, IL-15RA and CXCL9 (p<0.05). Higher peripheral Tfh (p<0.05), Tph (p<0.001) were also observed in the baseline flow cytometry result of AE patients compared to NAE patients. Based on these findings, predictive models for both irAEs and grade 2–4 irAEs were established, demonstrating good discriminatory ability.

**Conclusion:**

This study demonstrates that high-dimensional immune profiling can uncover novel blood-based immune signatures associated with the risk and mechanism of severe irAEs are effective biomarkers for predicting irAEs at both baseline and early stages of ICIs treatment.

## 1 Introduction

The advent of immune checkpoint inhibitors (ICIs) has tremendously transformed the therapeutic landscape for various malignancies, offering significant survival benefits to patients with gastrointestinal cancer (1–4). However, the excessive immune response elicited by ICIs can lead to irAEs, which constitute a group of autoimmune disorders affecting multiple organs or systems, including the skin, heart, liver, and musculoskeletal system (5, 6). According to large-scale population studies, the incidence of any type of irAEs ranges from 60% to 80% among patients who receive ICIs, with varied incidence across individual irAEs (7). The commonness and complexity of irAEs pose a challenge for clinicians in managing patients undergoing ICIs treatment. Exploration of predictive biomarkers and development of predictive models for irAEs represent essential areas of research. Identifying patients at higher risk for developing irAEs could enable personalized treatment regimens, preventing severe toxicities.

T cells are the primary targets of ICIs, and alterations in the phenotype or frequency of various T-cell populations may be linked to the development of irAEs. CD4+T cell subsets, including T helper cell (Th)1, Th2, and follicular helper T cells (Tfh), are essential for both effective anti-tumor immunity and immune self-tolerance (8, 9).

Tfh cells express surface molecules such as inducible T-cell co-stimulatory (ICOS), programmed cell death-1 (PD-1), and C-X-C chemokine receptor type 5 (CXCR5). They secrete the cytokine IL-21, which aids B cells in producing antibodies and differentiating into memory cells and plasma cells within germinal centers (10). The increase in Tfh cells has been implicated in various autoimmune diseases (11). Recent studies have provided growing evidence that ICIs treatment can promote a dysregulated Tfh cell response, leading to the development of irAEs (12). Studies in mice have shown that a deficiency of PD-1 in antigen-specific T cells results in an increased frequency of Tfh cells (13). Recent research has confirmed an increase in Tfh cells following anti-PD-1 treatment in vaccination, as observed in both cellular and transcriptomic analyzes (14). Additionally, recent studies have shown that interactions between Tfh and germinal center B cells are necessary for tumor control mediated by effector CD8+T cells (15–18). Moreover, a multi-omic analysis of over 18,000 patient samples further suggests a correlation between Tfh cells and irAEs (19).

Other T cell subsets with Tfh-like phenotype, including peripheral helper T cells (Tph) and follicular cytotoxic T cells (Tfc), also play essential role in autoimmune. Tph are also IL-21-producing T cells capable of localizing at inflammatory sites without CXCR5 expression. Tph cells have been shown to mediate B cell help through production of IL-21, and are believed to participate in many autoimmune diseases, including rheumatoid arthritis (RA), Sjögren’s syndrome (SS), and systemic lupus erythematosus (SLE) (20–22). Tfc cells are CD8+T cell subset having similar surface molecules to Tfh cells. Recent studies have shown that Tfc cells respond to immune checkpoint inhibition therapies that block PD-1 or PD-L1, which has significant implications for cancer immunology (23). Researches indicates that Tfc cells express effector molecules such as IFN-γ, maintain cytolytic function, and are associated with better prognoses in lung (24), colorectal (25), and pancreatic cancers (26). In our study, we defined Tph and Tfc cells as Tfh-like cells.

Recent studies revealed that Th1 and Th2 cells can modulate the immune response against tumors and influence the efficacy of ICI treatment (27). Many studies point to the intricate roles of helper T cell subsets in irAEs, suggesting that irAEs may be driven by newly activated CD4+ helper T cells (28). Th1 and Th2 cells are two key subsets of Th cells. In previous studies, Th1 cell overactivation was considered a primary event in many organ-specific autoimmune diseases, while Th2 cells play a significant role in allergic inflammatory diseases (29). Th1 and Th2 cells maintain a delicate balance through secretion of cytokines that cross-suppress each other (9). Imbalances in Th1 and Th2 cells have been identified as being involved in the development of malignant tumors, drug-induced immunotoxicity, and numerous inflammatory diseases (9, 30, 31). A prospective cohort study performed on 20 patients with arthritis-irAEs showed that a high frequency of Th1 cells was involved in the pathogenesis of ICIs related arthritis (32). Multiple pro-inflammatory cytokines, such as IL-12, are essential for the differentiation of Th1 cells and enhancing the cytotoxic activity of lymphocytes (33). Moreover, CD8+T cells primarily function as cytotoxic effector cells, but also express cytokine profiles analogous to CD4 subsets, termed cytotoxic T cells (Tc) 1 and Tc2 (34).

The distribution characteristics of peripheral T helper cells before and after early stages of ICI treatment, and their contributions to irAEs, remain poorly understood. Here, through immunophenotypic analysis, gene expression analysis, and clinical observation in gastrointestinal cancer patients, we find Tfh and Tph cells, as well as Th1/Th2 ratio, as potential biomarkers for predicting the occurrence of irAEs. Based on biomarkers above, we constructed a prediction model for irAEs occurrence and severity, and translate this model into a practical clinical tool, laying a foundation for clinical guidance for ICIs treatment.

## 2 Materials and methods

### 2.1 Patient enrollment

We sequentially recruited patients with unresectable tumors at the Department of Gastrointestinal Oncology in Peking University Cancer Hospital from December 1^st^, 2021, to December 1^st^, 2022. Eligible participants should have a clear pathological diagnosis of invasive gastrointestinal carcinoma, are going to receive anti-PD-1/PD-L1 therapies and meet the following criteria: 1) age ≥ 18; 2) Eastern Cooperation Oncology Group (ECOG) Performance Status is evaluated as 0 or 1; 3) with an expected survival ≥ 3 months; 4) naïve to immunotherapy including anti-PD-1/PD-L1 therapies. Patients who concurrently received other systemic treatments other than immunotherapy, including target therapy, radiotherapy, and cellular therapy were excluded. Patients with preexisting rheumatic diseases (e.g., SLE, RA, SS), hematological disorders (e.g., idiopathic thrombocytopenic purpura, myelodysplastic syndrome), and other disease conditions that require intervention with corticosteroids or immunomodulators were also excluded. Data on demographic characteristics, tumor type, treatment regimen, and irAEs type were recorded (**Table 1**). Both sexes were involved, and sex was not considered as a biological variable.

**Table 1:** Clinical characteristics of enrolled patients.

Characteristics	Patients with irAEs onset (n=22)	Patients without irAEs onset (n=29)
Age, years, mean ± SD	57.32 ± 11.42	57.62 ± 16.92
Gender		
Male	16	20
Female	6	9
BMI, mean ± SD	21.99 ± 2.97	21.72 ± 2.40
Tumor type		
Gastric cancer	14	18
Colorectal cancer	0	8
Neuroendocrinal cancer	5	2
Others	3	1
Treatment regimen		
Anti-PD-1/PD-L1	5	13
Anti-PD-1+anti-CTLA-4	14	10
Anti-PD-1+chemotherapy	3	6
Best treatment response		
Complete/partial response	11	14
Stable disease	6	11
Progressive disease	5	4
Highest irAEs grade		
1	9	
2	9	
3	2	
4	2	
IrAEs grade (total events of irAEs)		
1	22	
2	10	
3	2	
4	2	
Affected organ & systems (total events of any grade irAEs/grade 2–4 irAEs)		
Skin	12/2	
Musculoskeletal system	3/2	
Cardiovascular system	1/1	
Liver	6/2	
Gastrointestinal tract	2/2	
Thyroid	8/2	
Adrenal gland	1/0	
Pancreas	2/2	
Bone marrow	1/1	

Highest irAEs grade, number of patients with the highest-grade irAEs at each severity level; irAEs grade: total number of irAEs events per grade across all patients, recurrent events of the same type are not double-counted; others, other gastrointestinal cancers including esophageal cancer and carcinoma of small intestine; SD, standard deviation; irAEs, immune-related adverse events; BMI, body mass index. Skin: including rash; Musculoskeletal system: including myositis; Cardiovascular system: including myocarditis; Liver: including immune hepatitis, elevated liver enzymes, liver dysfunction; Gastrointestinal tract: including colitis; Thyroid: including hypothyroidism and hyperthyroidism; Adrenal gland: including adrenal insufficiency; Pancreatic: including pancreatic dysfunction and elevated blood amylase; Bone marrow: including myelosuppression.

### 2.2 Peripheral blood sample collection

For all patients, EDTA-anticoagulated whole blood was collected. Peripheral blood lymphocytes (PBMCs) were isolated by Ficoll-Hypaque density gradient centrifugation and immediately processed for RNA extraction and flow cytometry. The serum was separated, collected, and stored at -80°C for future proteomic analysis. In terms of sample collecting timing, three time points were adopted: 1) within 3 days before the first dose of immunotherapy (baseline); 2) 6 weeks after ICIs treatment (early stages); 3) when irAEs started if irAEs developed during the follow-up;

### 2.3 Patient follow-up and irAEs grading

All patients were followed up for 6 months. IrAEs status and grading were evaluated according to Common Terminology Criteria for Adverse Events (CTCAE) version 5.0. Uniform and broadly applicable definitions for irAEs terminology have not been established yet. In this study, any adverse event evaluated with an immunological basis that occurred within 6 months of treatment initiation was deemed as irAEs.

### 2.4 RNA sequencing and data analysis

TRizol was used to extract total RNA from PBMCs, and then mRNA was purified by poly-T oligo-attached magnetic beads. Fragmentation, double-strand cDNA synthesis, RNA degradation, overhangs clip, 3’ ends adenylation, and adaptor assembly were performed per protocol. The library fragments were purified with the AMPure XP system (Beckman Coulter, Beverly, USA) to select cDNA fragments of preferentially 370~420 bp in length. After PCR amplification, the PCR product was further purified by AMPure XP beads to generate the final library. Qubit 2.0 Fluorometer and qRT-PCR were used to quantify the library, and Agilent 2100 Bioanalyzer was employed to detect the insert size. Qualified libraries were then pooled and sequenced by the Illumina NovaSeq 6000 in a Sequencing by Synthesis manner, generating 150bp paired end reads.

Raw data were initially processed to generate clean data by removing adaptors, reads containing N base, and low-quality reads. Hisat2 was used to map clean reads to the reference genome (GRCh38.p13). FeatureCounts was employed to count the reads and to calculate the Fragments Per Kilobase of transcript sequence per Millions base pairs sequenced (FPKM) values. DESeq2 was used to perform the differential gene expression analysis. The p-values were adjusted using the Benjamini & Hochberg method. P-value<0.05 was set as the threshold for significantly differential expression. GO, KEGG, Reactome, and GSEA enrichment analyzes were achieved through clusterProfiler and GSEA tools.

### 2.5 Flow cytometry

For staining T cells subsets, PBMCs were stained for 30 min in dark at 4°C with fluorophore-conjugated monoclonal antibodies listed in **Supplementary Table 1**. Relative proportions of T cell subset were analyzed by flow cytometry using Beckman and CytExpert software. The gating strategies and phenotypic characterization of lymphocyte subpopulations are shown in **Supplementary Figure 1** (35). After anti-PD-1/PD-L1 therapies, the surface marker PD-1 cannot be stained in PBMCs. Therefore, when analyzing the early stages PBMCs, we used the CD4^+^CXCR5^+^ICOS^+^ subset to represent Tfh cells, the CD4^+^CXCR5^-^ICOS^+^ subset to represent Tph cells, and the CD8^+^CXCR5^+^ICOS^+^ subset to represent Tfc cells. The percentages of the various T cell subsets reported in this study refer to their proportions relative to CD4+ T cells or CD8+ T cells.

### 2.6 Olink proteomics assays

Biomarkers in serum were profiled using a 96-plex Immuno-Oncology panel developed by Olink Proteomics (Sweden) and serviced by Shanghai Biotechnology Corporation. The average intra-assay coefficient of variability (%CV) for serum was 3%. Data were expressed as normalized protein expression (NPX) values, an arbitrary unit on a Log2 scale. Unlike precise biomarker concentrations, NPX values represent relative quantification. Biomarkers with more than 60% of values below the level of detection (LOD) were excluded from further analyzes.

### 2.7 Prediction model construction

Baseline flow cytometry results were utilized for risk score development. Patients were randomly assigned to either the primary cohort (n=38) or the validation cohort (n=13) at a 3:1 ratio. 12 variables were entered into the selection process, including age, sex, BMI, CD8+T cells, CD4+T cells, Tph, Tfh, Tfc, Th1, Th2, Tc1, and Tc2 cells. The ‘lrm’ function from the ‘rms’ package in R was used to construct logistic regression models. We employed internal bootstrap validation to derive the Concordance Index (C-index) and Area Under the Curve (AUC) of the ROC curves to assess the performance of models. The performance of the internally validated models was tested in the validation cohort.

### 2.8 Statistical analysis

In all comparisons, participants have matched demography characteristics and treatment regimens. Descriptive analysis was employed to present demographic characteristics. Continuous variables were compared using t-tests for normally distributed data or Mann-Whitney tests for non-normally distributed data. Statistical significance was set at two-sided p-values < 0.05. Analyzes were performed using SPSS 24.0, Prism 9.0, and R (Version 4.3.2, https://www.r-project.org).

## 3 Results

### 3.1 Characteristics of patients

**Figure 1:**
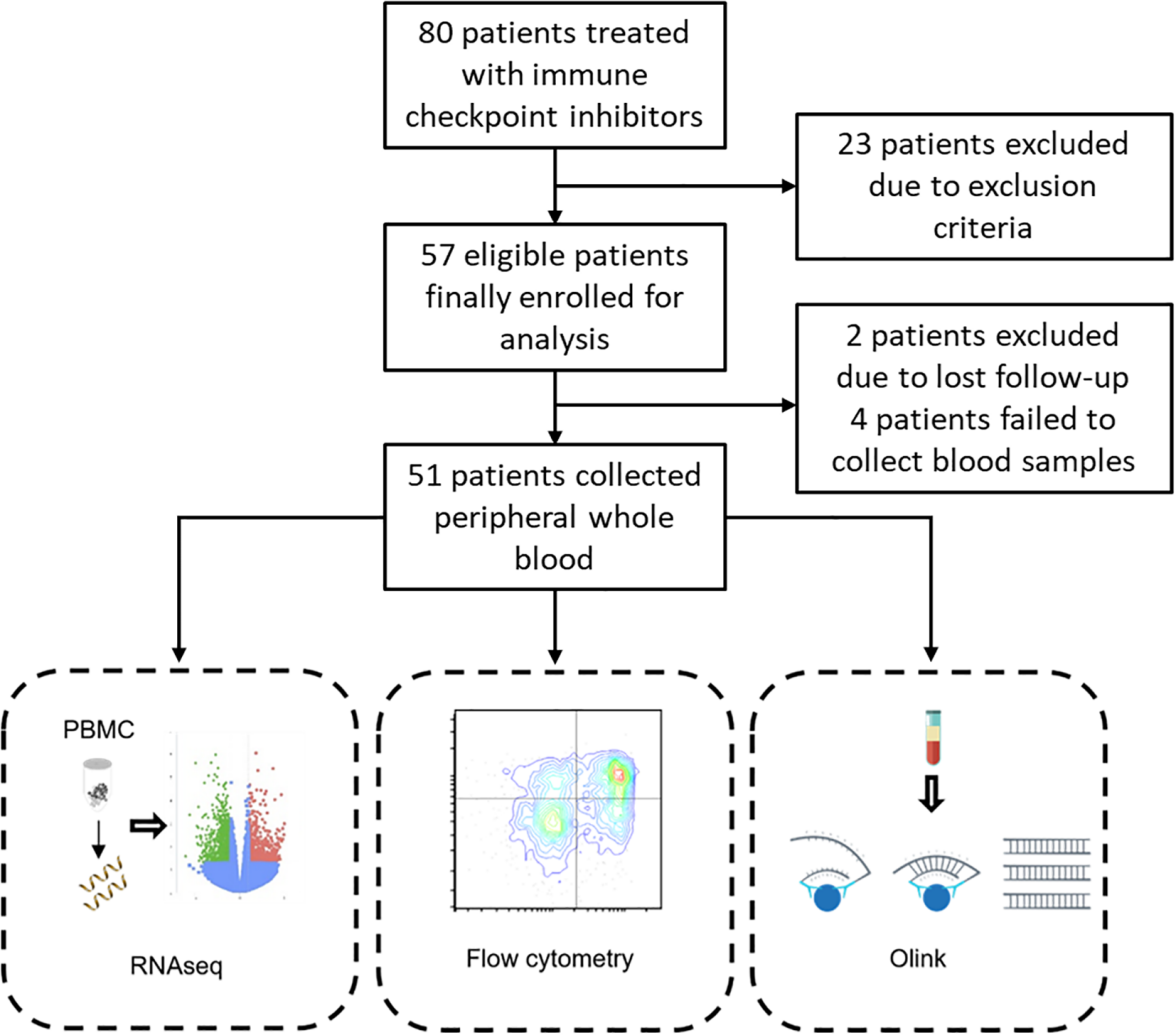
Study design and patient disposition.

In this prospective cohort study, 51 patients with gastrointestinal cancer were finally enrolled (**Figure 1**), including 32 (62.75%) patients with gastric, 8 (15.69%) patients with colorectal, 7 (13.73%) patients with neuroendocrinal cancer, and 4 (7.84%) patients with other gastrointestinal cancers. In terms of treatment regimens, 18/51 (35.3%) patients received anti-PD-1 or anti-PD-L1 monotherapy, 24/51 (47.06%) patients received anti-PD-1 and anti-CTLA-4 combinational therapy, and 9/51 (17.65%) patients received anti-PD-1 therapy combined with chemotherapy (**Table 1**). Twenty-two patients developed irAEs while 29 patients did not develop irAEs during the subsequent follow-up. These irAEs affected different organs and systems, including skin, muscles, heart, liver, gastrointestinal tract, endocrine system, and bone marrow. A total of 36 irAEs were recorded, including 14 grade 2–4 irAEs (**Table 1**). We categorized patients who developed irAEs during the follow-up into the AE group, and patients who didn’t develop irAEs into the NAE group.

Patients who received anti-PD-1 and anti-CTLA-4 combinational therapy had a higher incidence of irAEs compared to those who received either anti-PD-1, anti-PD-L1, or anti-PD-1 therapy combined with chemotherapy (**Table 1**). Notably, we ensured that the proportion of patients who received anti-PD-1 and anti-CTLA-4 combinational therapy was balanced between AE and NAE groups in the following analyzes. The characteristics of the patients and the specific analyses in which they were included are detailed in **Supplementary Table 1**

### 3.2 Multiple preexisting pro-inflammatory proteins characterized AE patients at baseline

**Figure 2:**
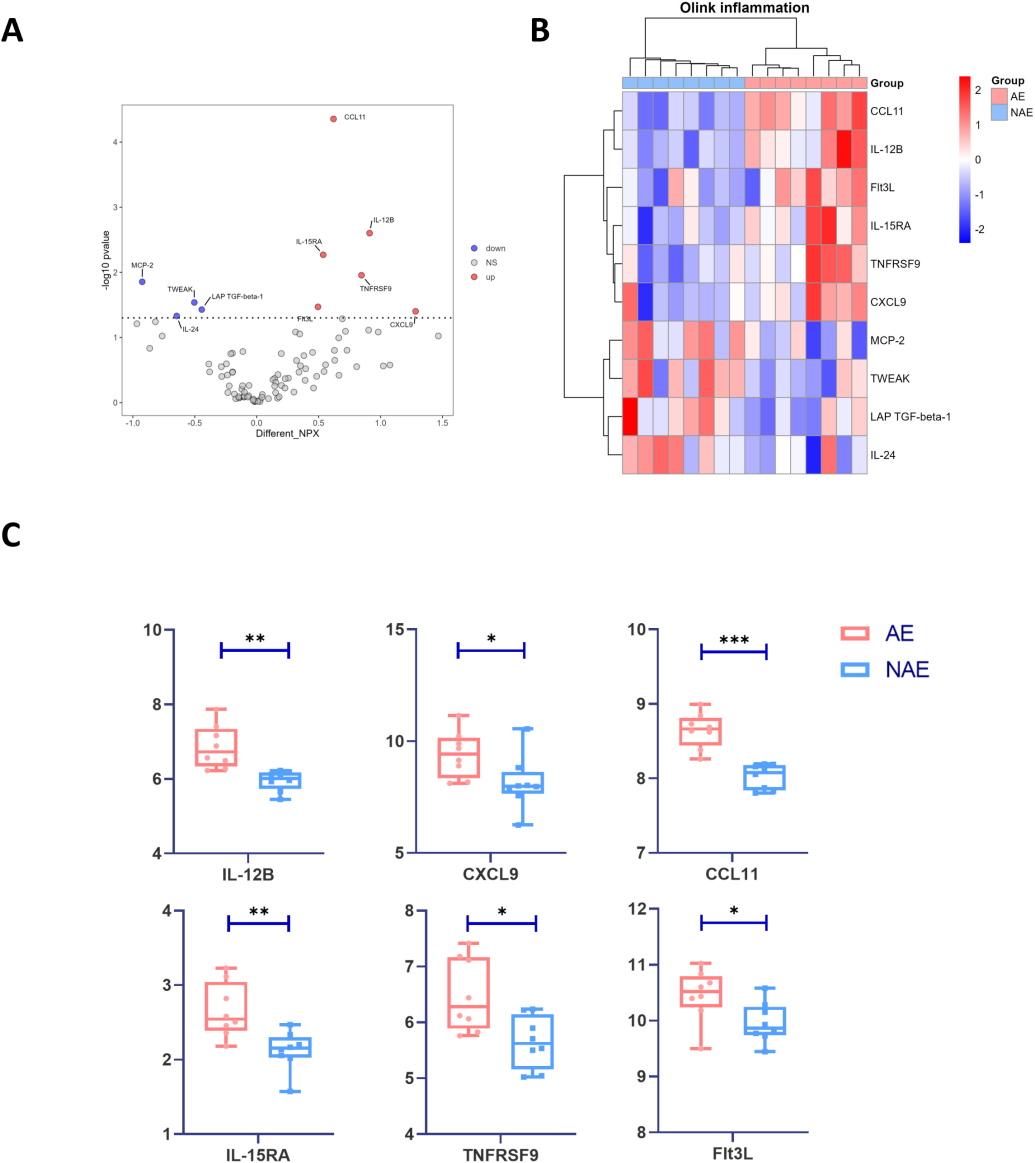
Cytokine profiling via Olink Target 96 inflammation panel. Olink technology was performed on serum from AE patients (n=8) and NAE patients (n=8). **(A)** Volcano plot highlighting the differential proteins (p < 0.05) between serum of AE and NAE patients with at baseline. The statistical analysis is determined by Student’s t-test. **(B)** Clustering heatmap for the differential proteins (p < 0.05) between serum of AE and NAE patients with at baseline. **(C)** Expression of differential protein in AE and NAE patients at baseline. The statistical analysis is determined by Student’s t-test. IrAEs, immune-related adverse events; AE, patients who developed irAEs within follow-up period after ICIs treatment; NAE, patients who didn’t develop irAEs within follow-up period after ICIs treatment; *p<0.05, **p<0.01, ***p<0.001.

We leveraged the Olink technology to quantify circulating inflammatory proteins in baseline serum samples from 8 AE patients and 8 NAE patients. Six proteins were significantly higher in AE patients at baseline (**Figures 2A, B**), including CCL11, IL-12β, IL-15RA, TNFRSF9, CXCL9, and Flt3L (p<0.05) (**Figure 2C**). Highly expressed inflammatory proteins in AE patients are particularly involved in helper T cells’ differentiation and function. IL-12β is a subunit of IL-12 that is essential in promoting Th1 cell differentiation and enhancing CD8+ T cell activity (33, 36). CXCL9 is an IFN-γ-induced chemokine that facilitates the migration of Th1 cells to sites of inflammation and infection and mediates Th1-mediated immune reactions. IL-15RA, a subunit of the IL-15 receptor, could enhance the effector functions of Th cells. These results suggest that AE patients exhibit a pre-inflammatory immune environment at baseline (37). IrAEs could be correlated with the proliferation and heightened functionality of cytotoxic T cells and Th cells, especially Th1 cells, within this environment.

### 3.3 Transcriptomic features of PBMCs in patients with irAEs after early stages of ICIs treatment

**Figure 3:**
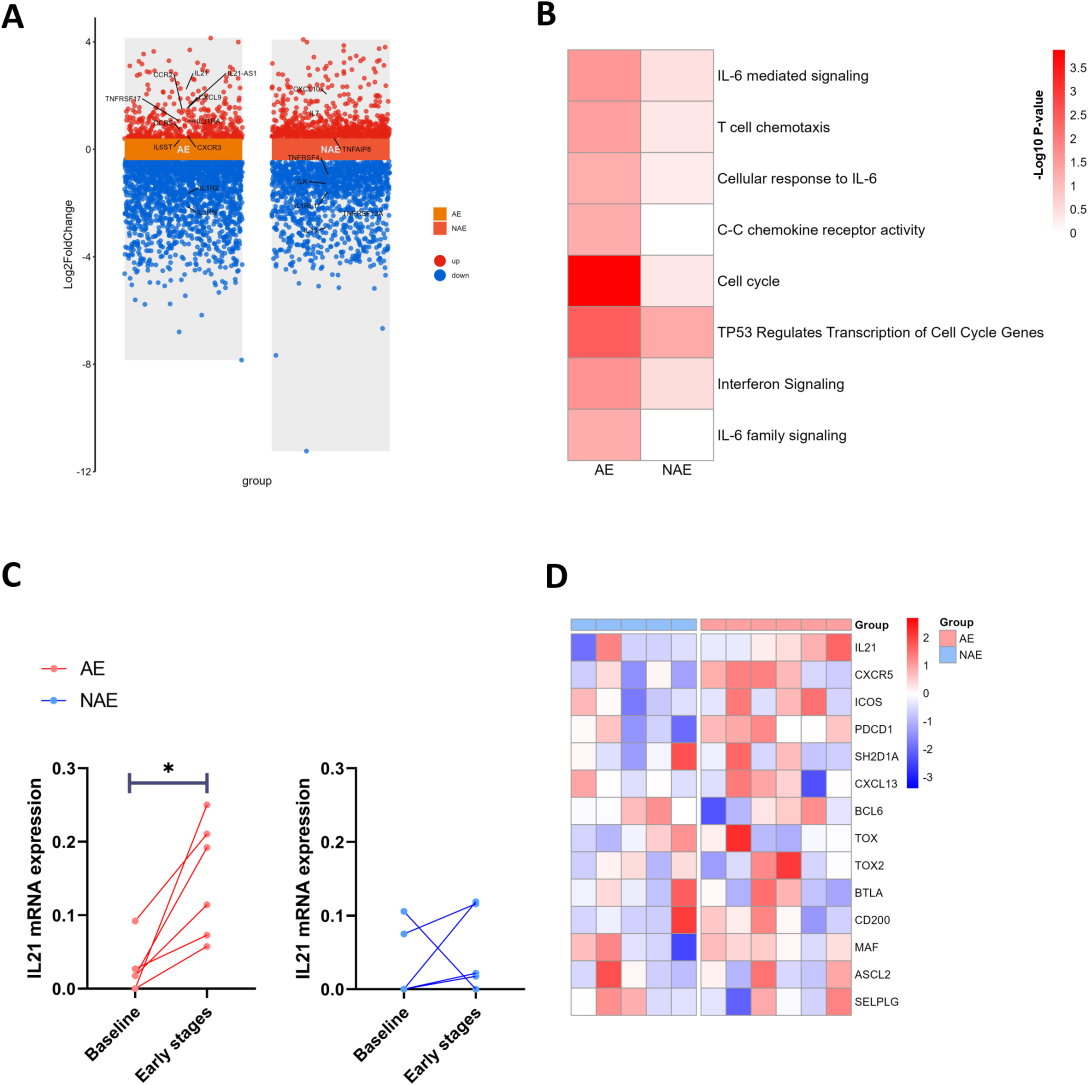
AE patients have a higher increase in Tfh-related genes after early stages ICIs treatment. **(A)**Volcano plot highlighting the up-regulated and down-regulated genes after early stages of ICI treatment in AE (n=6) and NAE (n=5) patients. **(B)** Representative GO, KEGG and Reactome biological process and pathways enriched in upregulated DE gene after early stages of ICI treatment in AE and NAE patients. **(C)** Dynamic of *IL-21* gene expression for AE (n=6) and NAE (n=5) patients after early stages of ICI treatment. Statistical significance was determined by Wilcoxon rank-sum test. **(D)** Heatmap for the change of Tfh-related gene expression after early stages of ICI treatment of PBMCs in AE (n=6) and NAE (n=5) patients. IrAEs, immune-related adverse events; AE, patients who developed irAEs within follow-up period after ICIs treatment; NAE, patients who didn’t develop irAEs within follow-up period after ICIs treatment; early stages, 6 weeks after receiving ICIs treatment. *p<0.05.

Using bulk RNA-seq, we characterized the transcriptomic features of PBMCs from 6 AE patients and 5 NAE patients at both baseline and after early stages of ICIs treatment. In AE patients, genes involved in inflammation, chemotaxis, and positive immune regulation, including *IL-21*, *IL21-AS1*, *IL6ST*, *IL31RA*, *CCR2*, *CCR5*, *CXCL9*, and *CXCR3*, were upregulated, while genes encoding for the antagonists of pro-inflammatory cytokine receptors, including *IL1R2*, *IL1RN*, were down regulated (**Figure 3A**). For NAE patients, such transcriptomic features were not observed. Gene Ontology (GO) and KEGG enrichment analyzes showed that upregulated differentially expressed (DE) genes in AE patients were significantly enriched in several immune activation pathways, including T cell activation, T cell migration, and proinflammatory cytokine pathways. These pathways were not revealed to be enriched in NAE patients (**Figure 3B** and **Supplementary Figures 2A, B**). These results suggest that ICIs treatment may induce stronger inflammatory activation and increased lymphocyte migration in AE patients compared to NAE patients.

We further visualized the top 50 gene features for 6 AE patients through heatmap at baseline and after early stages of ICIs treatment. *IL21* was among the top upregulated gene in AE patients after early stages of ICIs treatment (**Supplementary Figure 3**). *IL21* encodes IL-21, which is a signature cytokine of Tfh-like cells and plays a crucial role in ICIs-based immunotherapy (15, 16) and autoimmune diseases (21). *IL21* was upregulated after early stages of ICIs treatment (p<0.05), whereas such upregulation was not seen in NAE patients (**Figure 3C**). The dynamic changes of signature genes regarding Tfh cell after early stages of ICIs treatment were shown in heatmap (**Figure 3D**) (17, 21, 23, 38). AE patients displayed a stronger upregulation of Tfh-related genes after early stages of ICIs treatment. These results indicate that AE patients exhibit a characteristic increase in gene expression in PBMCs during the early stages of ICIs treatment, including genes associated with T cell activation, pro-inflammatory pathways, and Tfh-like cells. These genes may play a potential role in the development of irAEs.

### 3.4 Elevated peripheral Tfh-like cell and Th1/Th2 ratio after early stages ICIs treatment are associated with the development of irAEs

**Figure 4:**
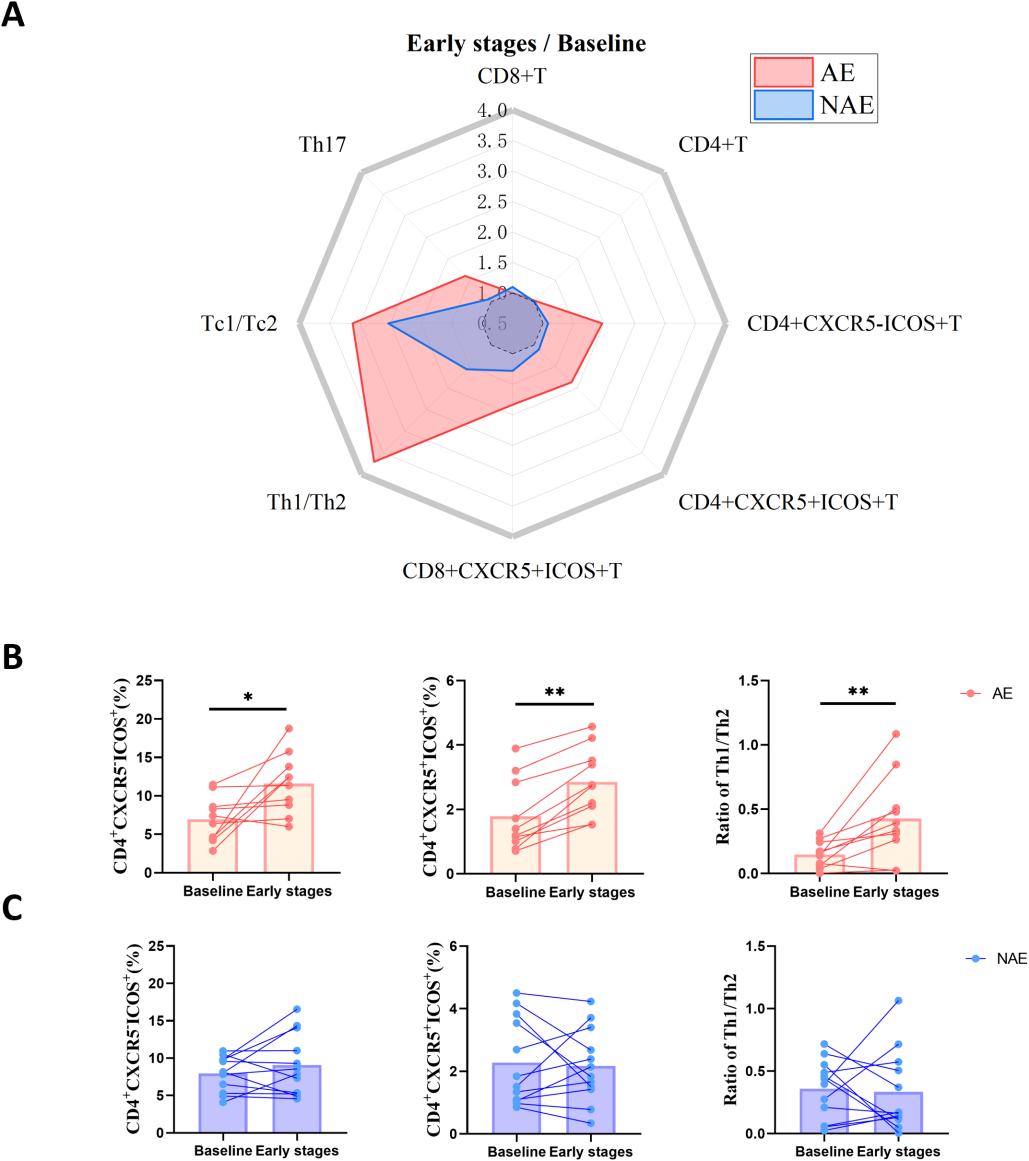
AE patients have a higher increase in Tfh-like cells and Th1/Th2 ratios after early stages ICIs treatment. **(A)** Radar maps of ratio of percentage of T cell subsets between early stages and baseline in AE (n=10) and NAE (n=12) patients. **(B)** Dynamic of percentage of CD4+T cell subsets between early stages and baseline in AE patients(n=10). Statistical significance was determined by Wilcoxon rank-sum test. **(C)** Dynamic of percentage of CD4+T cell subsets between early stages and baseline in NAE patients (n=12). Statistical significance was determined by Wilcoxon rank-sum test. IrAEs, immune-related adverse events; AE, patients who developed irAEs within follow-up period after ICIs treatment; NAE, patients who didn’t develop irAEs within follow-up period after ICIs treatment; CD4+CXCR5+ICOS+ subset represent Tfh cells; CD4+CXCR5-ICOS+ subset represent Tph cells; Tfh, follicular helper T cell; Tph, peripheral helper T cells; Tfc, follicular cytotoxic T cells; Th, helper T cells; Tc, cytotoxic T lymphocytes; early stages, 6 weeks after receiving ICIs treatment. *p<0.05, **p<0.01.

We utilized flow cytometry to analyze relative proportions of T cell subset in baseline and early stages PBMCs from 10 AE patients and 12 NAE patients. After early stages of ICIs treatment, AE patients exhibited increases in several T cell populations, including CD4^+^CXCR5^-^ICOS^+^ cells, CD4^+^CXCR5^+^ICOS^+^ cells, CD8^+^CXCR5^+^ICOS^+^ cells, Th17 cells, Th1/Th2 ratio and Tc1/Tc2 ratio (**Figure 4A**; **Supplementary Figure 4A**). Notably, the increases in CD4^+^CXCR5^-^ICOS^+^ cells (p<0.05), CD4^+^CXCR5^+^ICOS^+^ cells (p<0.01) and Th1/Th2 ratio (p<0.01) reached significant difference (**Figure 4B**). In contrast, these T cell subsets did not show significant elevation in NAE patients after early stages of ICIs treatment (**Figure 4C**; **Supplementary Figure 4A**). In addition, the expansion extent of CD4^+^CXCR5^+^ICOS^+^ cells (p<0.05) and Th1/Th2 ratio (p<0.05) also differed significantly between AE and NAE patients (**Supplementary Figure 4B**). These results suggest that AE patients displayed a more pronounced expansion of Tfh and Tfh-like cells, accompanied by a Th1-skewed phenotypic transformation. This indicates that the proportions of these T cell subsets may play a critical role in the development of irAEs and could serve as early predictors of irAEs during ICIs treatment.

### 3.5 Genes related to T cell activation and cytotoxicity are highly expressed in AE patients before receiving ICIs treatment

**Figure 5:**
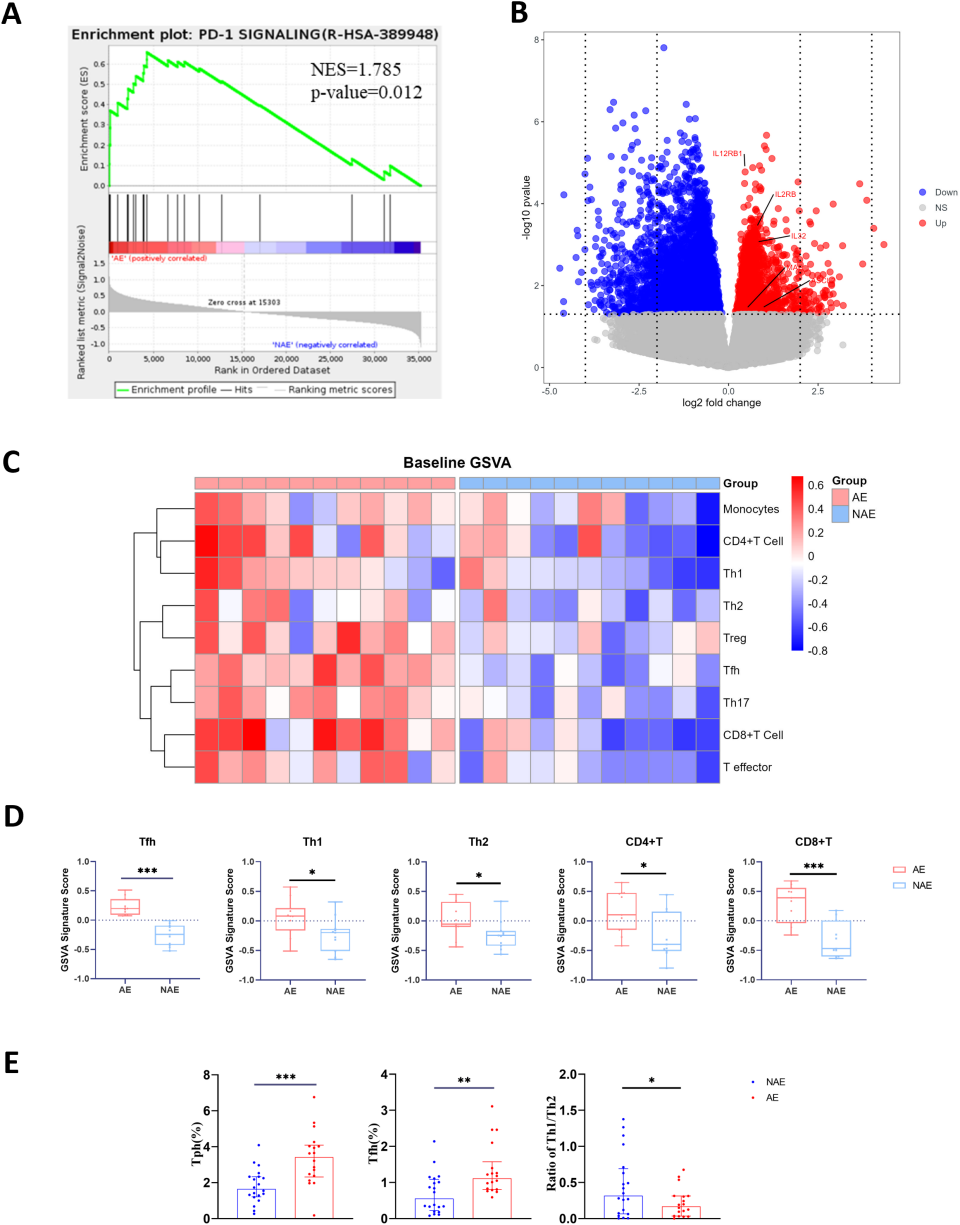
AE patients have a higher level of Tfh and Tfh-like cell at baseline. RNA-seq data of baseline PBMCs from AE (n=11) and NAE (n=11) patients were analyzed. **(A)** GSEA for the potential function of DE mRNAs of AE’s PBMCs at baseline. **(B)** Volcano plot highlighting the DE genes between 11 AE and 11 NAE patients at baseline. **(C)** Supervised whole-genome microarray analysis of 11 AE and 11 NAE patients. **(D)** Pathway analysis of Tfh, Th1, Th2, CD8+T cell, and CD4+T cell genes using GSVA showing significant differences in z-scores of each pathway in 11 AE and 11 NAE patients. The statistical analysis was determined by Student’s t-test. Flow cytometric analysis was performed on PBMCs from AE and NAE patients. **(E)** Comparison of the percentage of T cell subsets between AE (n=18) and NAE (n=20) patients at baseline. The statistical analysis was determined by Student’s t-test. IrAEs, immune-related adverse events; AE, patients who developed irAEs within follow-up period after ICIs treatment; NAE, patients who didn’t develop irAEs within follow-up period after ICIs treatment; Tfh, follicular helper T cell; Tph, peripheral helper T cells; Th, helper T cells; Treg, regular T cells. GSEA, gene set enrichment analysis; GSVA, gene set variation pathway analysis. The statistical analysis is determined by Student’s t-test. *p<0.05, **p<0.01, ***p <0.001.

RNA-seq data of baseline PBMCs from 11 AE patients and 11 NAE patients were analyzed, indicating distinct proinflammatory transcriptomic signatures in AE patients. For upregulated DE genes in AE patients, GO, KEGG and Reactome enrichment analysis showed an enrichment in pathways involved in inflammation, immune cell chemotaxis, and positive regulation of the immune system, particularly T cell activation and cytotoxicity (**Supplementary Figure 5**). Gene set enrichment analysis (GSEA) indicated that the gene set PD-1 signaling was enriched in AE patients (NES=1.785, p=0.012) (**Figure 5A**). Th1-related genes and Tfh-related genes, including *IL12RB1*, *IL2RB*, *MAF* and *ASCL2*, were highly expressed in AE patients at baseline (**Figure 5B**). These results suggest that AE patients exhibit stronger T cell activity compared to NAE patients at baseline.

We also performed gene set variation pathway analysis (GSVA) (21). A list of genes defining the gene signatures is provided in **Supplementary Table 2**. Unsupervised clustering showed that AE patients clustered differently from NAE patients (**Supplementary Figure 6**). Supervised clustering showed significant upregulations in AE patients compared to NAE patients in Tfh (p<0.001), Th1 (p<0.05), Th2 (p<0.05) (**Figures 5C, D**). Additionally, genes related to CD4+ T cells (p<0.05) and CD8+ T cells (p<0.001) were significantly upregulated in AE patients (**Figures 5C, D**).

Flow cytometry of baseline PBMCs from 18 AE patients and 20 NAE patients confirms that AE patients have a significantly higher level of Tph (p<0.001) and Tfh (p<0.01) at baseline compared to NAE patients (**Figure 5E**), suggesting that the high frequency of Tfh and Tph cells in AE patients at baseline is attributable to heightened differentiation within the subset itself, rather than an overall expansion of total T cells. The level of Th1/Th2 ratio (p<0.05) was significant lower at baseline compared to NAE patients (**Figure 5E**). Due to the low expression levels of Tfc cells, we used the CD8^+^CXCR5^+^ICOS^+^ subset as a proxy for comparison. However, no significant difference was observed (**Supplementary Figure 7**). The differences of Th17 cell and Tc1/Tc2 ratio were not significant across the two groups either (**Supplementary Figure 7**). These findings confirmed the value of Tfh, Tph, and the Th1/Th2 ratio as early predictors of irAEs through both cellular and transcriptomic analyses.

Further stratification of AE patients into grade 2-4 (n=10) and grade 1 (n=8) irAEs subgroups demonstrated a consistent trend: patients with grade 2–4 irAEs exhibited higher levels of Tfh and Tph cells, accompanied by lower Th1/Th2 ratios compared to those with grade 1 irAEs, although these differences did not achieve statistical significance due to the limited sample size (**Supplementary Figure 8**). These findings suggest that these T cell subsets not only serve as predictors for irAEs occurrence but also exhibit a potential correlation with irAEs severity.

### 3.6 Construction of the risk score prediction model for irAEs

Logistic regression models were constructed to predict irAEs risk and moderate to severe irAEs risk, based on the following predictors: Age, Tph, Tfh, and Th1/Th2 ratio. Flow cytometry results from 38 patients at baseline were included in primary cohort and the other 13 patients were included in validation cohort (**Supplementary Table 1**). Among primary cohort, 20 were NAE patients and 18 were AE patients (10 had irAEs with a highest grade of 2-4, 8 had irAEs with a highest grade of 1). Among validation cohort, 9 were NAE patients and 4 were AE patients (3 had irAEs with a highest grade of 2-4, 1 had irAEs with a highest grade of 1) (**Supplementary Table 1**).

**Figure 6:**
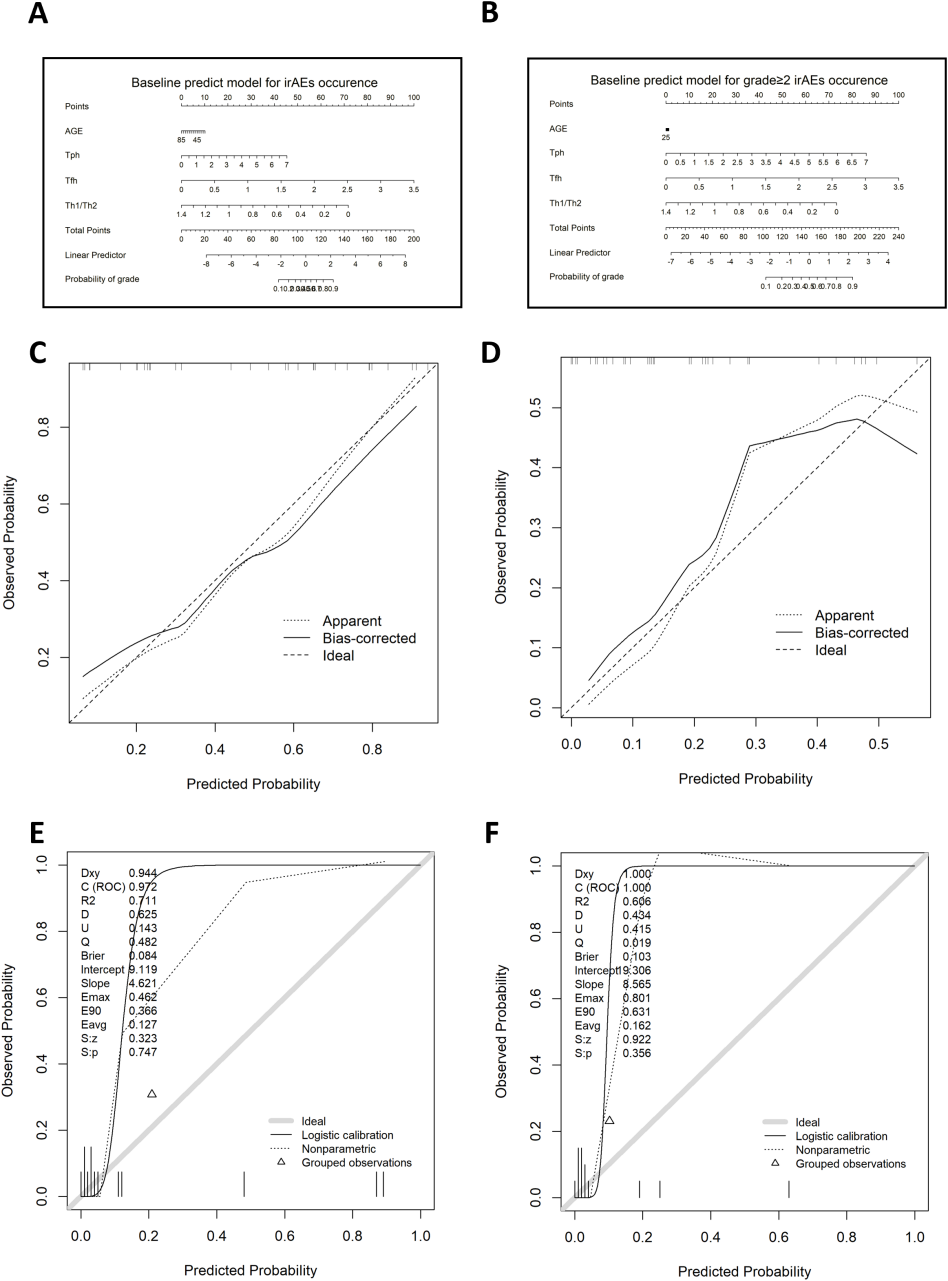
The clinical tools for predicting irAEs occurrence and severity basing on T cell subsets at baseline. **(A)** Nomograms for the prediction of irAEs occurrence based on baseline flow cytometry results. **(B)** Nomogram for the prediction of grade 2–4 irAEs occurrence based on baseline flow cytometry results. **(C)** Calibration curve of the nomogram for the prediction of irAEs occurrence based on baseline flow cytometry results in the primary cohort. **(D)** Calibration curve of the nomogram for the prediction of grade 2–4 irAEs occurrence based on baseline flow cytometry results in the primary cohort. **(E)** Calibration curve of the nomogram for the prediction of irAEs occurrence based on baseline flow cytometry results in the validation cohort. **(F)** Calibration curve of the nomogram for the prediction of grade 2–4 irAEs occurrence based on baseline flow cytometry results in the validation cohort. IrAEs, immune-related adverse events. Tfh, follicular helper T cell; Tph, peripheral helper T cells; Th, helper T cells.


The model for predicting the probability of irAEs and grade of 2–4 irAEs was developed and presented as the formulas and the nomograms (**Figures 6A, B**):

 Probability of irAEs=e^ −1.953−0.016·Age+0.606·Tph+2.668·Tfh−4.789·Th1/Th21+e^ −1.953−0.016·Age+0.606·Tph+2.668·Tfh−4.789·Th1/Th2Low−risk:≤ 0.229;Medium−risk:> 0.229 and≤ 0.653;High−risk:>0.653

Probability of grade 2−4 irAEs=e^ −3.683+0.001·Age+0.604·Tph+1.404·Tfh−2.573·Th1/Th21+e^ −3.683+0.001·Age+0.604·Tph+1.404·Tfh−2.573·Th1/Th2Low−risk:≤0.105;Medium−risk:>0.105 and≤0.28;High−risk:>0.28

Each factor was assigned a predicting score, and the sum of 4 scores was localized on the axis of total points, suggesting the prediction of the probability of irAEs occurrence.

Additionally, the predict model based on age and the dynamic of Tfh, Tph and Th1/Th2 ratio after early stages was built based on the flow cytometry results from 22 patients (10 AE patients and 12 NAE patients), with formulas and nomograms provided as well (**Supplementary Figure 9**). However, due to the limited sample size, we were unable to build the model for predicting the occurrence of grade 2–4 irAEs.

### 3.7 Apparent performance of the prediction models in the primary cohort

For the model predicting irAEs occurrence based on baseline T cell subsets level, the C-index was 0.822, and the R² was 0.394, with an AUC of 0.875 (95% confidence interval: 0.765–0.985) (**Supplementary Figure 10A**). The calibration curve showed good concordance between predicted and observed outcomes in the primary cohort (**Figure 6C**). The Hosmer-Lemeshow test produced a nonsignificant result (p=0.769), indicating no deviation from a perfect fit. For the model predicting grade 2–4 irAEs based on baseline T cell subsets level, the C-index was 0.784, and the R² was 0.261, with an AUC of 0.846 (95% confidence interval: 0.717–0.976) (**Supplementary Figure 10B**). Limited by the small sample size of patients with grade 2–4 irAEs, the calibration curve demonstrated slightly poorer agreement compared to the previous model (**Figure 6D**). However, the Hosmer-Lemeshow test yielded a nonsignificant result (p=0.296), suggesting that this model also showed no evidence of misfit. These metrics indicate that both models exhibit good predictive power and calibration, making them valuable for clinical applications in predicting both the occurrence and the severity of irAEs.

For the model predicting irAEs occurrence based on the dynamic of T cell subsets level after early stages of ICIs treatment, the C-index was 0.679 with an R² of 0.141, along with an AUC of 0.808 (95% confidence interval: 0.595–1.000) (**Supplementary Figure 9**). However, due to the limited sample size, the calibration curve of this model did not exhibit satisfactory consistency.

### 3.8 Independent validation

Good calibration was observed for the probability of irAEs occurrence in the validation cohort (**Figure 6E**). The Hosmer-Lemeshow test yielded a nonsignificant statistic (p=0.330), and the C-index was 0. 972 (95% CI, 0.895 to 1). However, limited by the small sample size of patients with grade 2–4 irAEs, the probability of grade 2–4 irAEs occurrence demonstrated less robust calibration (**Figure 6F**). Nonetheless, the C-index for this model was 0.867 (95% CI, 0.721–1), suggesting good discriminative ability despite the small cohort size.

### 3.9 Clinical use through web-based calculate

**Figure 7:**
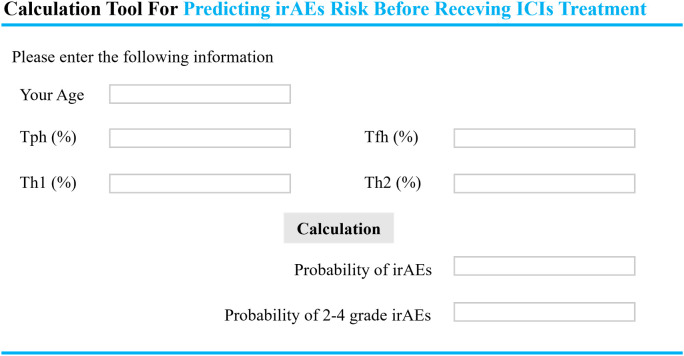
The clinical tools for predicting irAEs occurrence and severity. (https://small-yifanwang.github.io/irae-risk-prediction/). IrAEs, immune-related adverse events. Tfh, follicular helper T cell; Tph, peripheral helper T cells; Th, helper T cells.

To translate these analytical advancements into practical clinical tools, we developed a user-friendly Graphical User Interface (GUI) (**Figure 7**). This interface enables clinicians to input a patient’s age and the ​percentages of Tfh, Tph, Th1, and Th2 subsets within CD4^+^T cells, thereby generating predictive estimates for both the overall probability of irAEs and the probability of grade 2–4 irAEs. The GUl embodies the essence of our predictive algorithm and provides a simplified and accessible interface for real-time applications in clinical settings. It provides decision-making guidance for the choice of ICIs treatment for patients with gastrointestinal cancers. This software, designed for local use, is built on the sklearn library in Python and incorporates a PyQt5 graphical interface. It ensures both reliability and ease of use, making it well-suited for demanding healthcare environments.

## 4 Discussion

Identifying predictive biomarkers for irAEs is essential to personalize treatments and mitigate severe toxicities. CD4+ T helper cells, including Tfh, Tph, Th1 and Th2 cells, are crucial for immune response modulation (27, 28). Imbalances in these cells are linked to multiple autoimmune diseases (9, 20, 21, 29–31). Recent research highlights the role of Tfh and Th1/Th2 ratios in both anti-tumor response and irAEs (15–18, 32, 33), However, research on the T cell profiles of AE patients prior to receiving ICI treatment is limited, and comprehensive profiling and predictive models for irAEs are currently lacking.

Our findings illuminate the distinct circulating immunological landscape of irAEs in gastrointestinal cancer patients treated with ICIs. According to our results, after early stages of ICIs treatment, patients who developed irAEs exhibit a higher increase in proinflammatory gene features of PBMCs, accompanied by higher increase in Tfh, Tph cells, and Th1/Th2 ratio, indicating that these cells are potentially pathogenic in irAEs. Furthermore, patients who developed irAEs displayed a pre-existing inflammatory immune milieu at baseline, characterized by elevated circulating inflammatory proteins, upregulated T cell activation genes in PBMCs, higher baseline levels of Tfh and Tph cells levels, and a lower Th1/Th2 ratio. Notably, the baseline level of these T cell subsets also correlated with irAEs severity. These results underscore that patients who developed irAEs exhibit a proinflammatory state prior to ICIs treatment, accompanied by a distinct T cell profile. Based on these features, we developed a predictive model, which yields high efficiency in predicting the occurrence and severity of irAEs.

Regarding Th1 and Th2 cells, transcriptomic profiling revealed significantly elevated expression of Th1- and Th2-associated genes in AE patients compared to NAE patients at baseline. However, flow cytometric analysis failed to demonstrate a predominant Th2 differentiation bias. Given that RNA-seq detects early transcriptional changes while flow cytometry measures later-stage protein expression, we propose the following mechanistic interpretation: in AE patients, a subset of CD4+ T cells may have an enhanced propensity to differentiate into Th1 cells and upregulate Th1-associated genes. These pre-activated Th1 cells likely exhibit low CXCR3 expression before exposure to Th1-polarizing cytokines,such as IL-12. Following the immune activation induced by ICI treatment, these pre-activated Th1 cells rapidly differentiate into CXCR3+ Th1 cells (39, 40). This hypothesis aligns with Picelli et al.’s demonstration of RNA-seq’s exceptional sensitivity in capturing incipient transcriptional changes during early immune activation (41), thereby explaining the observed discordance between transcriptomic and proteomic profiling methodologies.

Tfh cells contribute to the maintenance of both humoral and cellular immunity. On the one hand, Tfh supports the development of tumor-associated tertiary lymphoid structures, promoting the differentiation and maturation of germinal center B cells through the CXCL13-CXCR5 signaling pathway (8, 42, 43). On the other hand, the interaction between Tfh and B cells could facilitate CD8 + T cell-mediated cytotoxicity. Interestingly, Tfh cells highly express membrane co-inhibitory molecules (PD-1, TIGIT) and co-stimulatory molecules (ICOS, CD40L), thus are functionally variable (8). Therefore, ICIs could potentially remodel the Tfh phenotype by blocking these immune co-inhibitory molecules. Alonson et al.’s study yielded that Tfh activation signatures (such as IL-21 and IL-4 secretion, CD38 expression, etc.) were significantly upregulated in patients who responded to ICIs (43). Herati et al. revealed that patients receiving anti-PD-1 treatment had a significant increase in circulating Tfh and plasmablasts. Our study further expands the conclusion, indicating that circulating Tfh is expanded in early stages of ICIs treatment, and is highly parallel to the occurrence of irAEs. We also observed that for ICI-treated patients who developed irAEs, *IL21* was expressed higher in PBMCs in early stages of treatment compared with baseline, indicating from another point of view that Tfh is involved in the development of irAEs.

The observation that these immunological changes were present before the onset of clinical irAEs suggests a predisposition in certain individuals towards developing these complications. The predisposition is caused by either baseline immune dysregulation or the specific immunological milieu created by the tumor and its microenvironment. The identification of predictive biomarkers, including specific T cell subsets and cytokine profiles, offers a promising avenue for preemptive identification of patients at higher risk for irAEs, enabling personalized approaches to ICIs treatment.

Our study provides insights into the immunological mechanisms driving irAEs and establishes clinically efficient predicting models of irAEs based on the relative abundance of circulating helper T cell subsets at baseline and in early stages of ICIs treatment. The models classified risk into low, moderate, and high categories, while individual risk stratification requires clinicians’ systematic evaluation of patient-specific factors, thereby integrating computational predictions with clinical expertise to guide personalized risk-benefit deliberations. Future research should focus on validating these findings in larger, prospective clinical studies and exploring the integration of predictive biomarkers into clinical practice to tailor ICIs treatment. The ultimate goal is to enhance the safety and efficacy of cancer immunotherapy, maximizing benefits for patients while minimizing the risk of adverse effects.

Although our study makes contributions to the optimization of ICIs treatment, there are several limitations. First, although we basically ensure the comparability of patient groups in each analysis, the relatively small cohort size and the heterogeneity of the patient population, including cancer types and treatment regimens, highlight the need for larger studies to validate our findings. Moreover, despite demonstrating strong discriminative ability, the irAEs prediction models based on this small cohort exhibits less robust calibration. Future studies should incorporate larger sample sizes to enhance model optimization. Second, even though CTCAE helps clinicians to formalize and standardize the diagnosis of irAEs, the identification and grading of these toxicities is largely dependent on the subjective judgment of physicians. Third, there is currently no evidence supporting our hypothesis regarding Th1 and Th2 cells at baseline; further deep sequencing of Th cell subsets to investigate their distribution could provide valuable insights into the mechanisms underlying irAEs. Fourth, regarding Th17 cells, our study did not observe significant intergroup differences comparable to those reported in other studies, which may be attributed to our relatively small cohort size. Future studies with larger cohorts are warranted to examine the association between irAEs occurrence and both baseline levels and early-stage dynamics of Th17 cells. Fifth, helper T cells were comprehensively profiled and investigated in this study, while other immune cells and circulating cytokines may also play roles in the development of irAEs, which is not the focus of our study but could potentially affect the efficacy of our current predicting model. Future studies should incorporate the functional analysis of other immune compartments that could potentially link with the development of irAEs.

## 5 Conclusion

Our study indicates that Tfh and Tph cell subsets are involved in the development of irAEs of gastrointestinal cancer patients and present opportunities for predicting the occurrence of irAEs before ICIs treatment. Understanding the immunological landscape that predisposes patients to irAEs is the prerequisite to achieving a more personalized cancer treatment approach. Future research should focus on expanding our understanding of the immune mechanisms, exploring the therapeutic potential of immune modulators, and ultimately, enhancing the safety and efficacy of ICIs treatment for cancer patients.

## Data Availability

The RNA-seq data presented in the study are deposited in the Sequence Read Archive (SRA) repository, accession number PRJNA1241479. Other original contributions presented in the study are included in the article’s Supporting Data Values. Further inquiries can be directed to the corresponding authors.
