# Supplementary material for: Baseline profile peripheral Tfh cells predict immune-related adverse events in immune checkpoint inhibitor therapy of gastrointestinal cancer
Source: Front Immunol. 2025 May 29;16:1559275. doi: 10.3389/fimmu.2025.1559275 (PMC12163322; doi:10.3389/fimmu.2025.1559275)
Supplement: Supplementary file 2 [file Image1.pdf]

Supplementary Appendix

This appendix has been provided by the authors to give readers additional information about their work.

Supplement to: Jing He, Zhi Peng, Yifan Wang, Zhening Zhang, et al. Baseline profile peripheral Tfh cells predict immune-related adverse events in immune checkpoint inhibitor therapy of gastrointestinal cancer.

Figure S1. Phenotypic characterization of T cell subsets by flow cytometry in human.

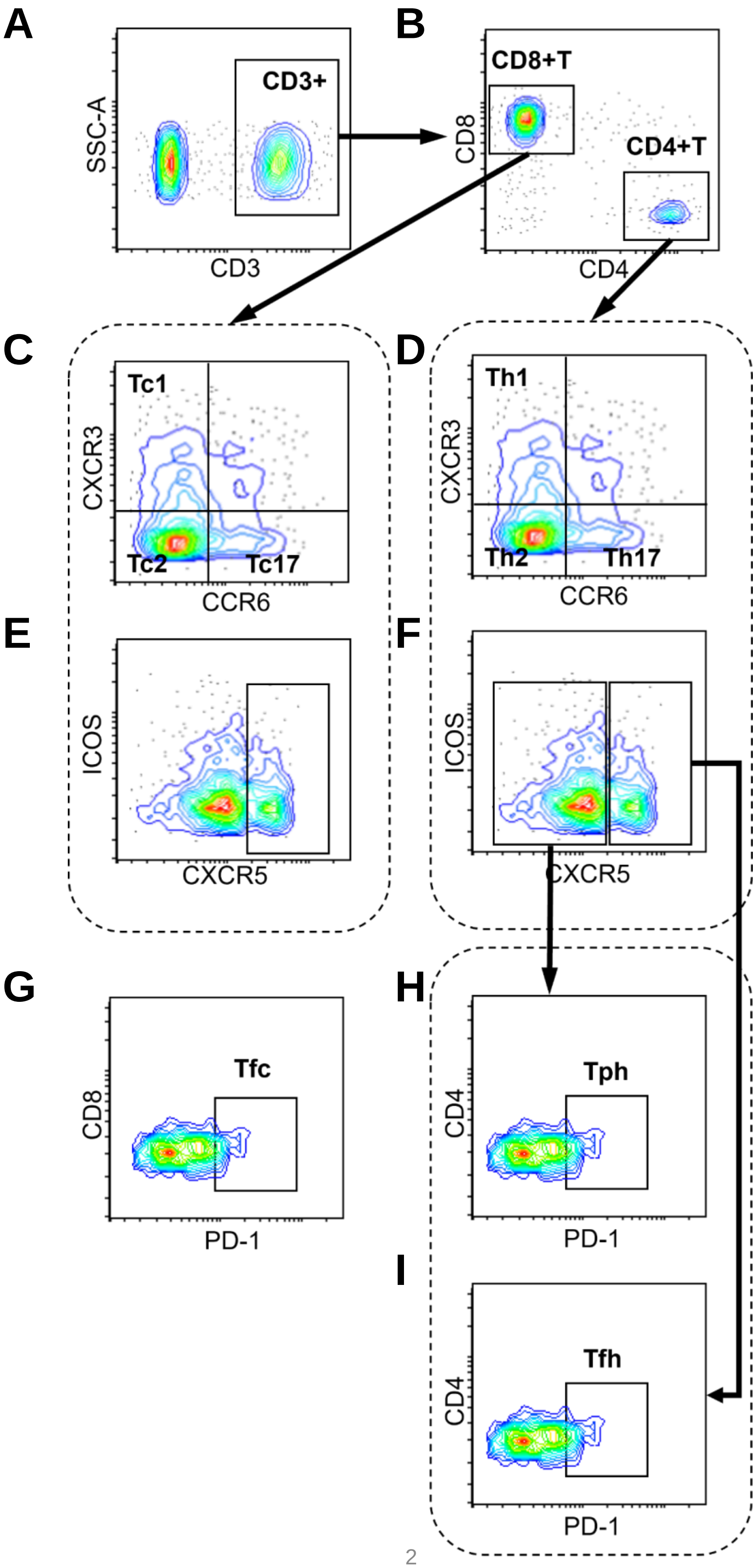

Figure S1. Phenotypic characterization of T cell subsets by flow cytometry in human.

Lymphocyte subsets	Phenotypic characterization
T cell	CD3+
Tfh	CD3+CD4+CXCR5+ICOS+PD-1+
Tph	CD3+CD4+CXCR5-ICOS+PD-1+
Tfc	CD3+CD8+CXCR5+ICOS+PD-1+
Th1	CD3+CD4+CXCR3+CCR6-
Th2	CD3+CD4+CXCR3-CCR6-
Th17	CD3+CD4+CXCR3-CCR6+
Tc1	CD3+CD8+CXCR3+CCR6-
Tc2	CD3+CD8+CXCR3-CCR6-
Tc17	CD3+CD8+CXCR3-CCR6+

Figure S1. Phenotypic characterization of T cell subsets by flow cytometry. Panel (A), T cell is identified as $CD3^{+}$ subset within lymphocytes. Panel (B), T cell is classified into $CD4^{+}$T cell and $CD8^{+}$ T cell. Panel (C), in $CD3^{+}CD8^{+}$ subset, Tc1, Tc2, and Tc17 cells were identified as $CXCR6^{-}CXCR3^{+}$ (Tc1), $CXCR6^{-}CXCR3^{-}$ (Tc2) and $CXCR6^{+}CXCR3^{-}$ (Tc17). Panel (D), in $CD3^{+}$ $CD4^{+}$ subset, Th1, Th2, and Th17 cells were identified as $CXCR6^{-}CXCR3^{+}$ (Th1), $CXCR6^{-}CXCR3^{-}$ (Th2) and $CXCR6^{+}CXCR3^{-}$ (Th17). Panel (E), $CD3^{+}CD8^{+}CXCR5^{+}ICOS^{+}$cells. Panel (F), $CD3^{+}CD4^{+}CXCR5^{+}ICOS^{+}$cells. Panel (G), Tfc cells were determined as $CD3^{+}CD8^{+}CXCR5^{+}ICOS^{+}$PD-1 $^{+}$ cells. Panel (H), Tph cells were determined as $CD3^{+}CD4^{+}CXCR5^{-}ICOS^{+}$ PD-1 $^{+}$ cells. Panel (I), Tfh cells were determined as $CD3^{+}CD4^{+}CXCR5^{+}ICOS^{+}$ PD-1 $^{+}$ cells. Tc, cytotoxic T cell. Th, helper T cells. Tfh, follicular helper T cells. Tph, T peripheral helper cells. Tfc, follicular cytotoxic T cells. ICOS: inducible costimulator; CCR6: C-C chemokine receptor type 6. CXCR3: C-X-C chemokine receptor type 3. CXCR5: C-X-C chemokine receptor type 5.

Figure S2. GO, KEGG and Reactome enrichment analyses for DE gene after early-stage of ICI treatment in AE and NAE patients.

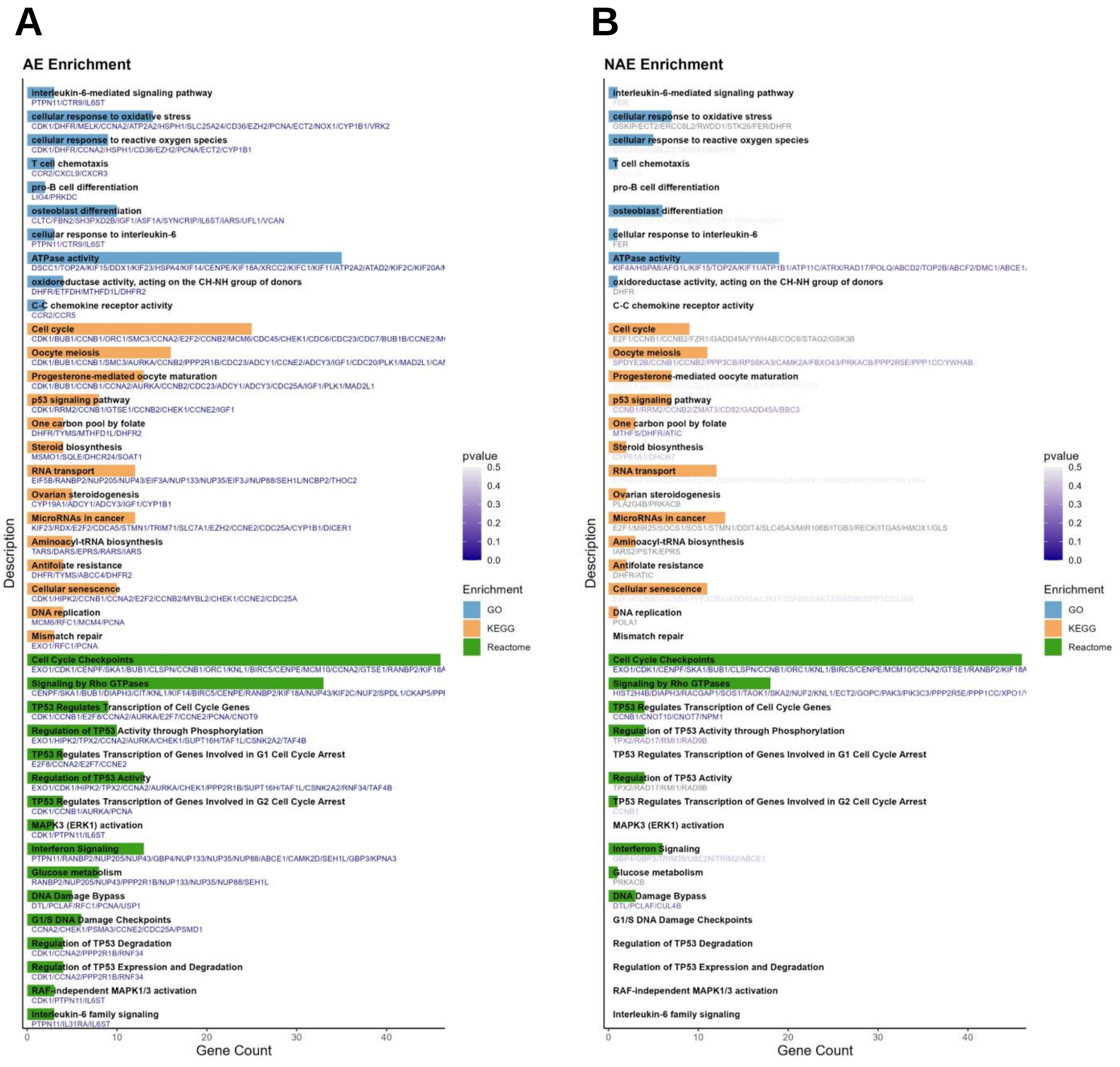

Figure S2. GO, KEGG and Reactome biological process and pathways enriched in upregulated DE gene after early stages of ICI treatment in AE (A) and NAE (B) patients. AE, patients who developed irAEs within follow-up period after ICIs treatment; NAE, patients who didn’t develop irAEs within follow-up period after ICIs treatment;

Figure S3. Heatmap of the top 50 features for 6 AE patients in early-stage and baseline in readcount.

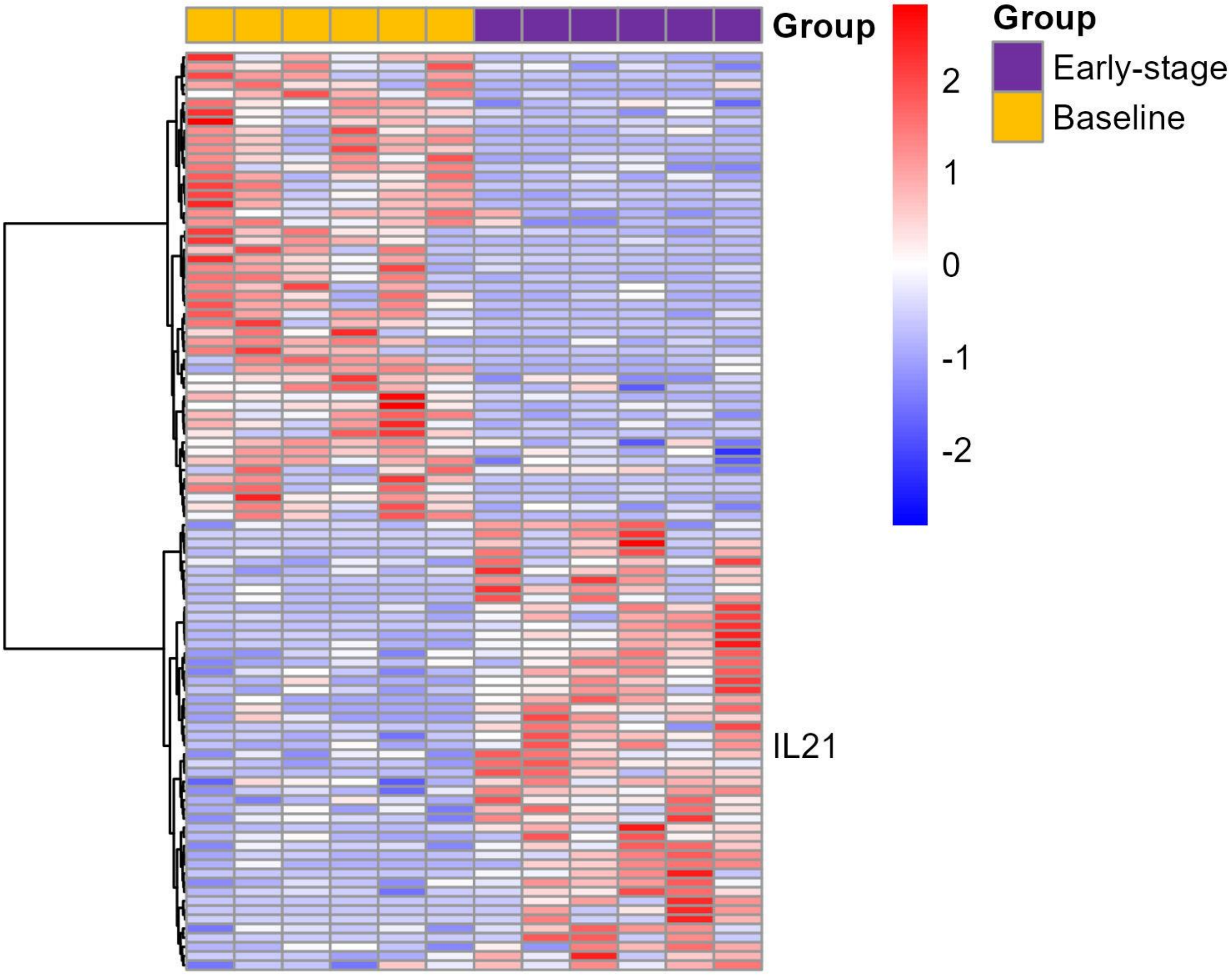

Figure S3. Heatmap of the top 50 features for 6 AE patients in early-stage and baseline in readcount. AE, patients who developed irAEs within follow-up period after ICIs treatment; Early stages, 6 weeks after receiving ICIs treatment;

Figure S4. Dynamic of percentage of T cell subsets between early-stage and baseline.

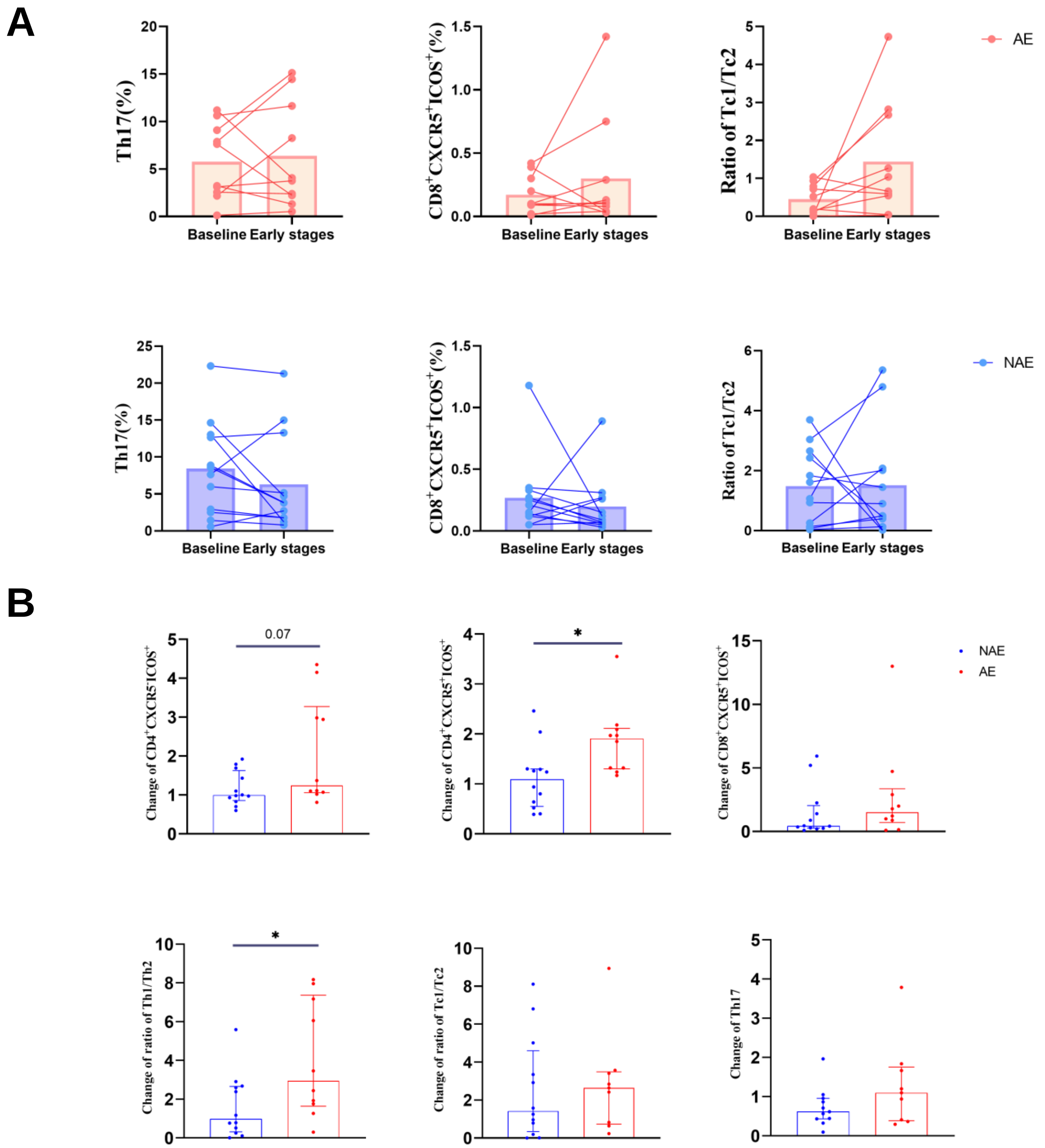

Figure S4. (A) Dynamic of percentage of Th17 and CD8$^+$ T cell subsets between early stages and baseline in AE and NAE patients. Statistical significance was determined by Wilcoxon rank-sum test. (B) Comparison of ratio of percentage of T cell subsets between early stages and baseline in AE and NAE patients. Statistical significance was determined by Mann-Whitney U test. AE, patients who developed irAEs within follow-up period after ICIs treatment. NAE, patients who didn't develop irAEs within follow-up period after ICIs treatment; CD4$^+$CXCR5$^+$ICOS$^+$ subset represent Tfh cells; CD4$^+$CXCR5$^-$ICOS$^+$ subset represent Tph cells; CD8$^+$CXCR5$^+$ICOS$^+$ subset to represent Tfc cells; Tfh, follicular helper T cell; Tph, peripheral helper T cells. Tfc, follicular cytotoxic T cells. Th, helper T cells; Tc, cytotoxic T lymphocytes; Early stages, 6 weeks after receiving ICIs treatment;

Figure S5. Dynamic of percentage of T cell subsets between early-stage and baseline.

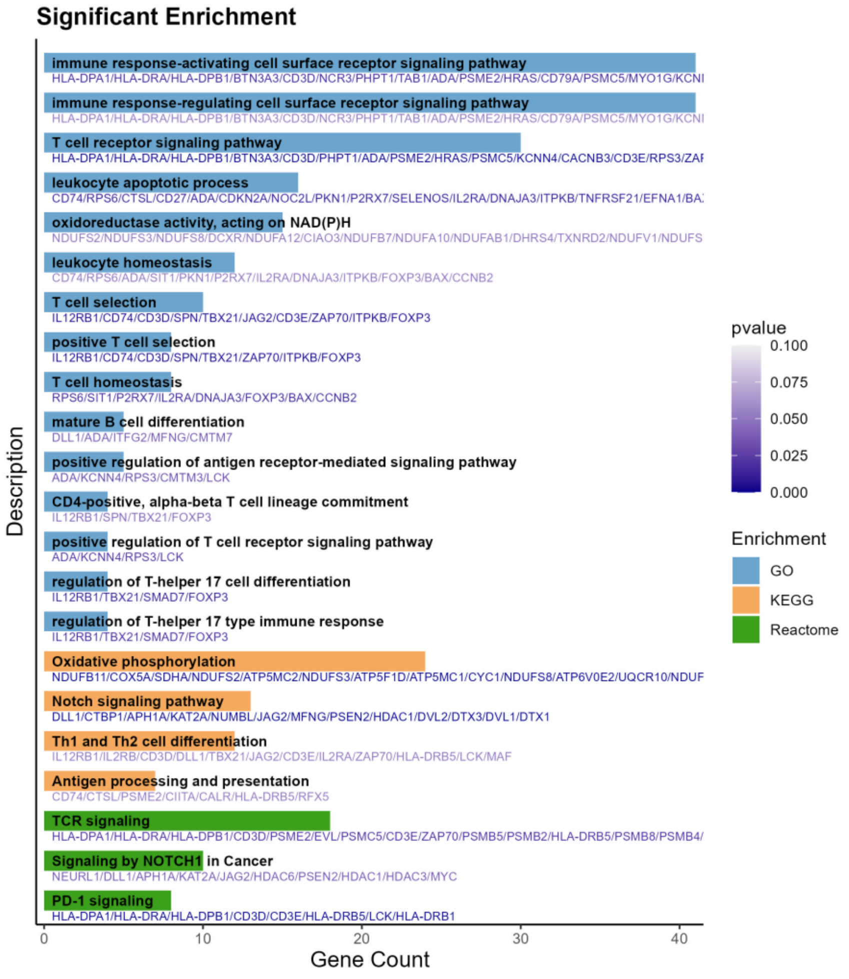

Figure S5. GO, KEGG and Reactome enrichment analysis for DE genes of AE patients at baseline. AE, patients who developed irAEs within follow-up period after ICIs treatment.

Figure S6. Supervised whole-genome microarray analysis of AE and NAE patients.

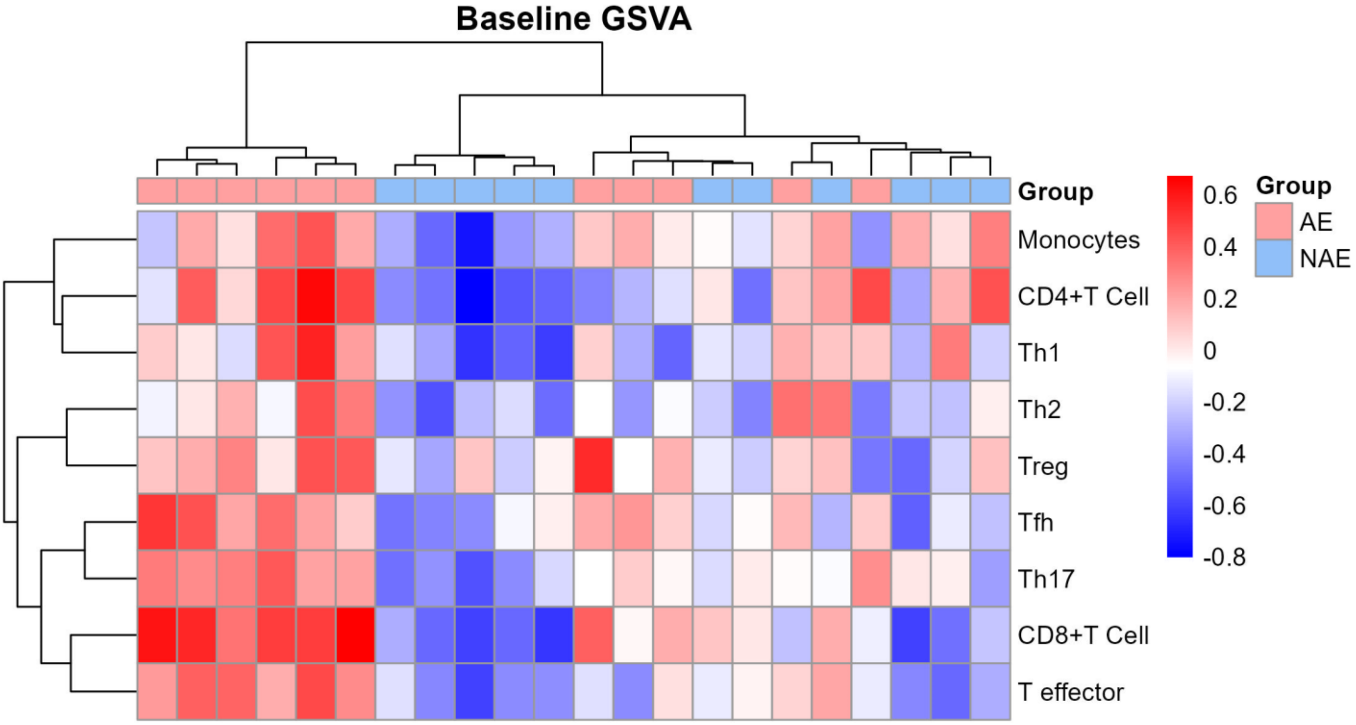

Figure S6. Supervised whole-genome microarray analysis of 11 AE and 11 NAE patients. AE, patients who developed irAEs within follow-up period after ICIs treatment; NAE, patients who didn't develop irAEs within follow-up period after ICIs treatment; Tfh, follicular helper T cell; Th, helper T cells.

Figure S7. Dynamic of percentage of T cell subsets between early-stage and baseline.

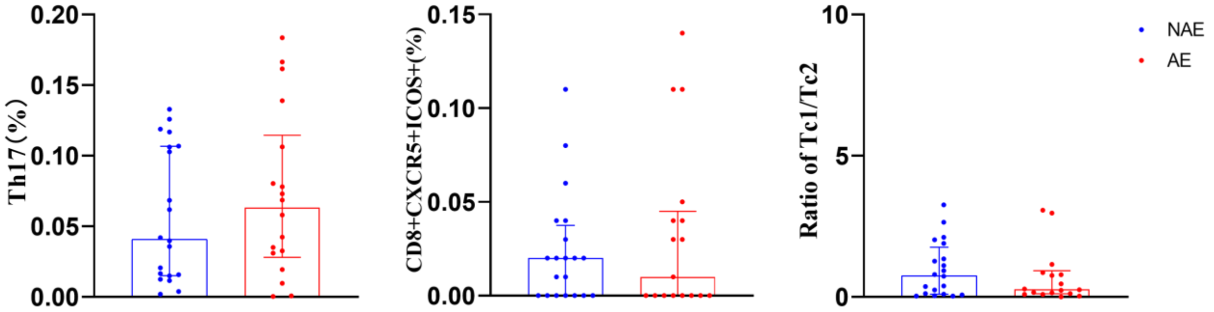

Figure S7. Comparison of the percentage of Th17 and CD8+ T cell subsets between AE (n=18) and NAE (n=20) patients at baseline. The statistical analysis was determined by Student's t-test. AE, patients who developed irAEs within follow-up period after ICIs treatment. NAE, patients who didn't develop irAEs within follow-up period after ICIs treatment; $CD8^{+}CXCR5^{+}ICOS^{+}$ subset to represent Tfc cells; Th, helper T cells; Tc, cytotoxic T lymphocytes.

Figure S8. The clinical tools for predicting irAEs occurrence and severity basing on T cell subsets at early stages.

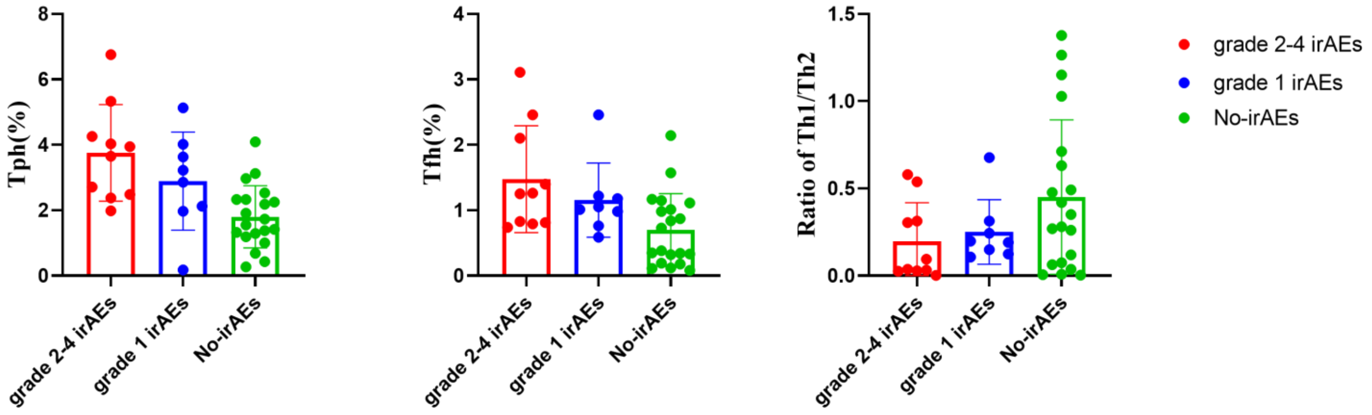

Figure S8. Comparison of the percentage of T cell subsets between grade 2-4 irAEs patients (n=10), grade 1 irAEs patients (n=8), and No-irAEs (n=20) patients at baseline. The statistical analysis was determined by Student's t-test. IrAEs, immune-related adverse events; grade 2-4 irAEs, patients who developed irAEs with a highest grade of 2-4; grade 1 irAEs, patients who developed irAEs with a highest grade of 1; No-irAEs, patients who didn’t develop irAEs within follow-up period after ICIs treatment; Tfh, follicular helper T cell; Tph, peripheral helper T cells; Th, helper T cells.

Figure S9. The clinical tools for predicting irAEs occurrence and severity basing on T cell subsets at early stages.

A

$$Probability\ of\ irAEs=\frac{exp(-4.871+0.022\cdot Age+1.383\cdot Tph+0.709\cdot Tfh+0.313\cdot Th1/Th2)}{1+exp(-4.871+0.022\cdot Age+1.383\cdot Tph+0.709\cdot Tfh+0.313\cdot Th1/Th2)}$$

B

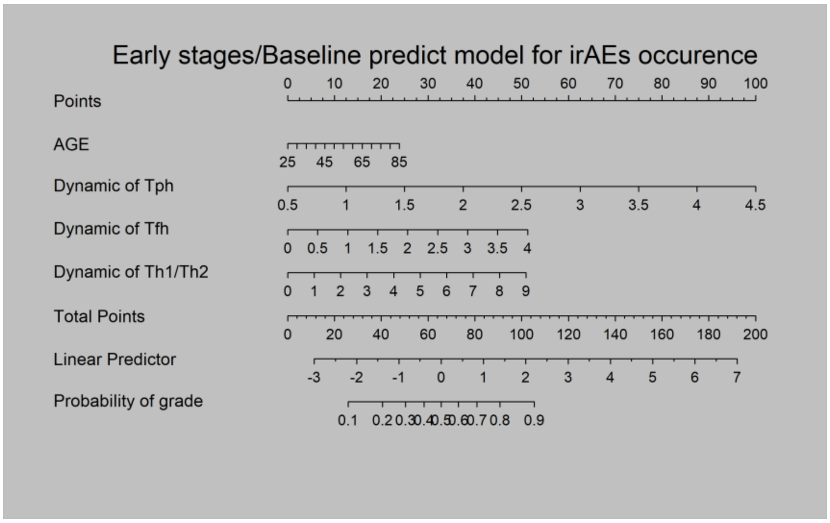

C

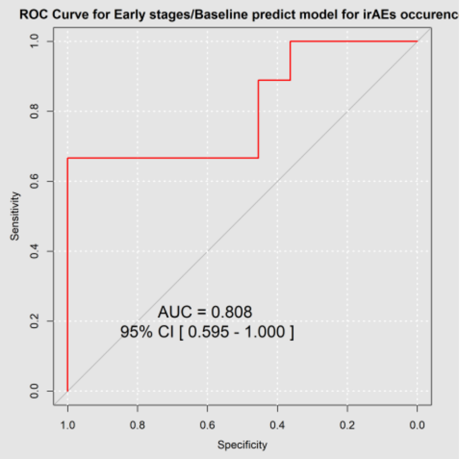

D

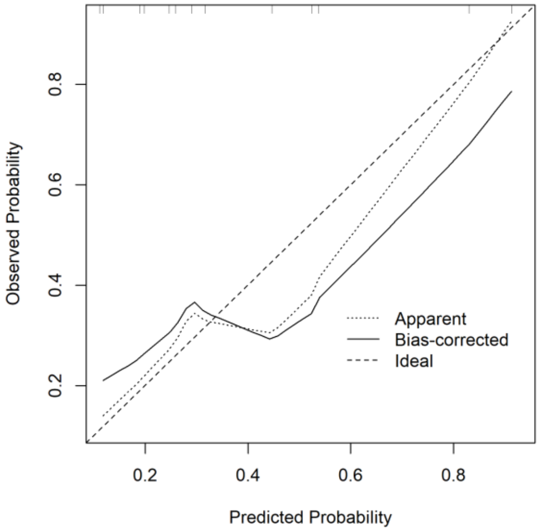

Figure S9. (A) Formulas for the logistic model to calculate the probability irAEs occurrence based on dynamic of flow cytometry results of 22 patients (10AE and 12 NAE) after early-stage of ICI treatment. (B) Nomograms for the prediction of irAEs occurrence based on dynamic of flow cytometry results. (C) The ROC of the nomogram for predicting irAEs occurrence based on dynamic of flow cytometry results. (D) Calibration curve of the nomogram for the prediction of irAEs occurrence based on dynamic of flow cytometry results in the primary cohort. AUC for the nomogram was 0.808 (95% CI 0. 595-1.000). irAEs, immune-related adverse events. Tfh, follicular helper T cell; Tph, peripheral helper T cells; Th, helper T cells; early stages, 6 weeks after receiving ICIs treatment.

Figure S10. ROC of the nomograms.

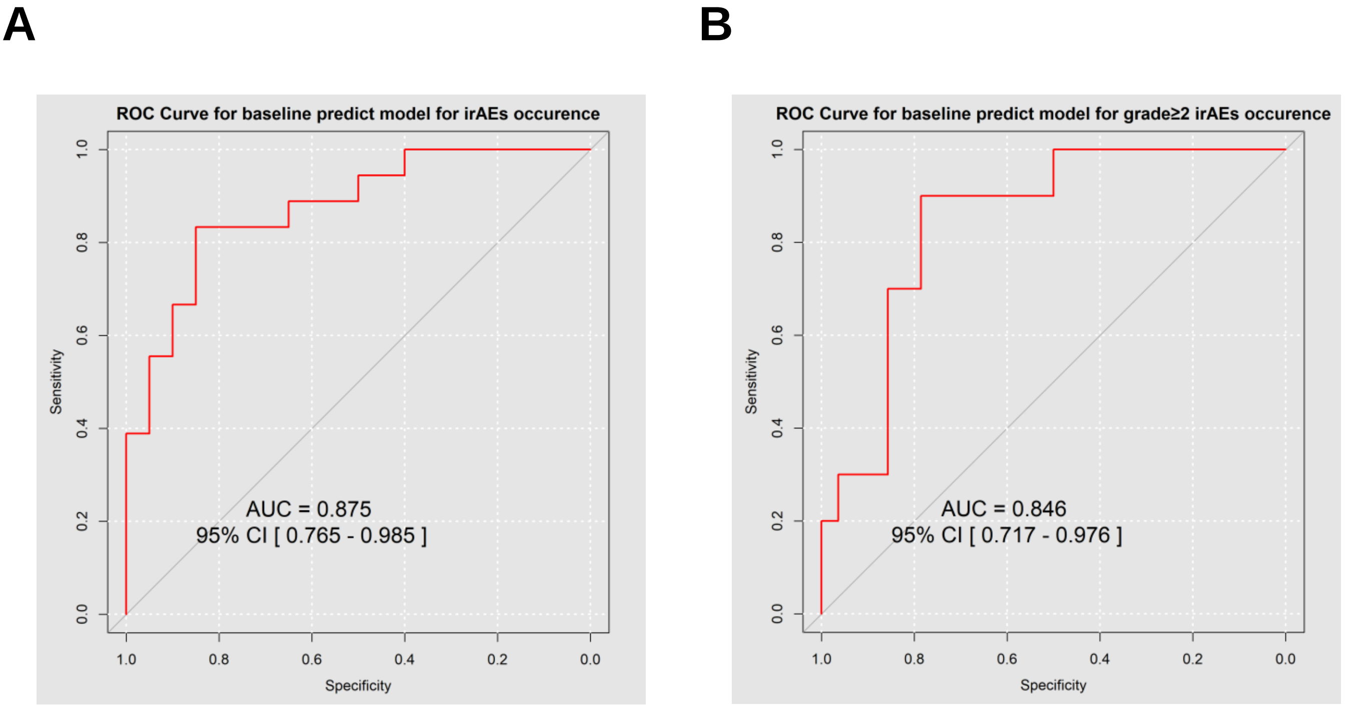

Figure S10. (A) The ROC of the nomogram for predicting irAEs occurrence based on baseline flow cytometry results. AUC for the nomogram was 0.875 (95% CI 0.765-0.985). (B) The ROC of the nomogram for predicting grade 2-4 irAEs occurrence based on baseline flow cytometry results. AUC for the nomogram was 0.846 (95% CI 0.717-0.976).
