# Supplementary material for: Baseline profile peripheral Tfh cells predict immune-related adverse events in immune checkpoint inhibitor therapy of gastrointestinal cancer
Source: Front Immunol. 2025 May 29;16:1559275. doi: 10.3389/fimmu.2025.1559275 (PMC12163322; doi:10.3389/fimmu.2025.1559275)
Supplement: Supplementary file 3 [file Image2.pdf]

Supplementary Material

This supplementary file presents the protocol-based re-stratification analyses of the data. All supplementary figure titles correspond to their respective main figures.

Fig 3

C

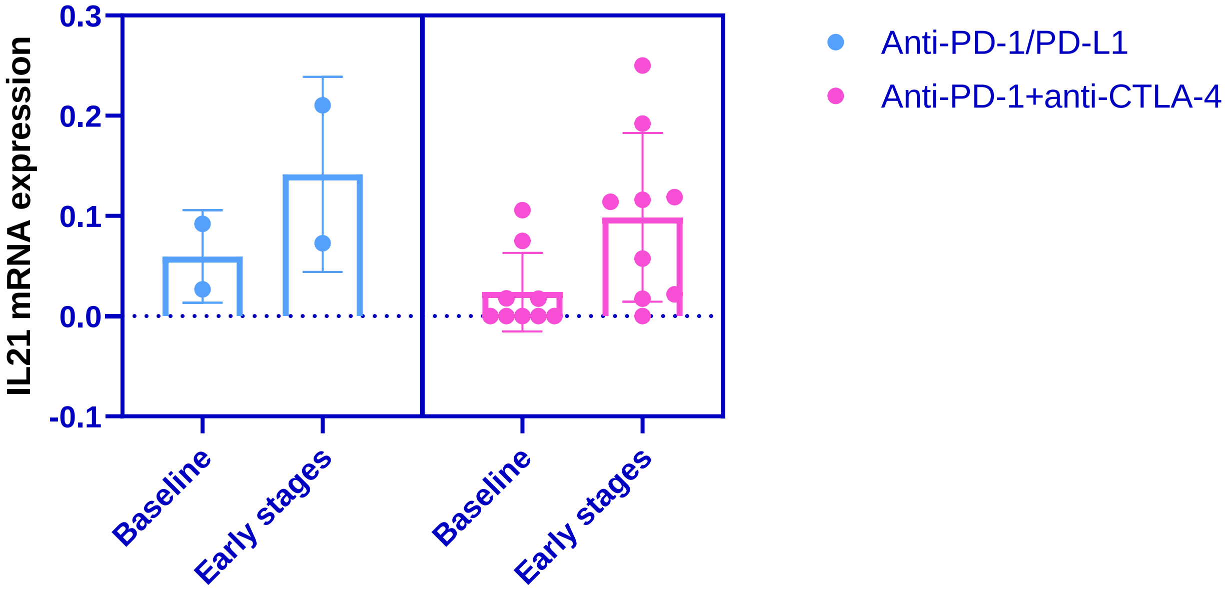

IL21 mRNA expression
0.3
0.2
0.1
0.0
-0.1
Baseline
Early stages
Baseline
Early stages
Anti-PD-1/PD-L1
Anti-PD-1+anti-CTLA-4

Fig 4

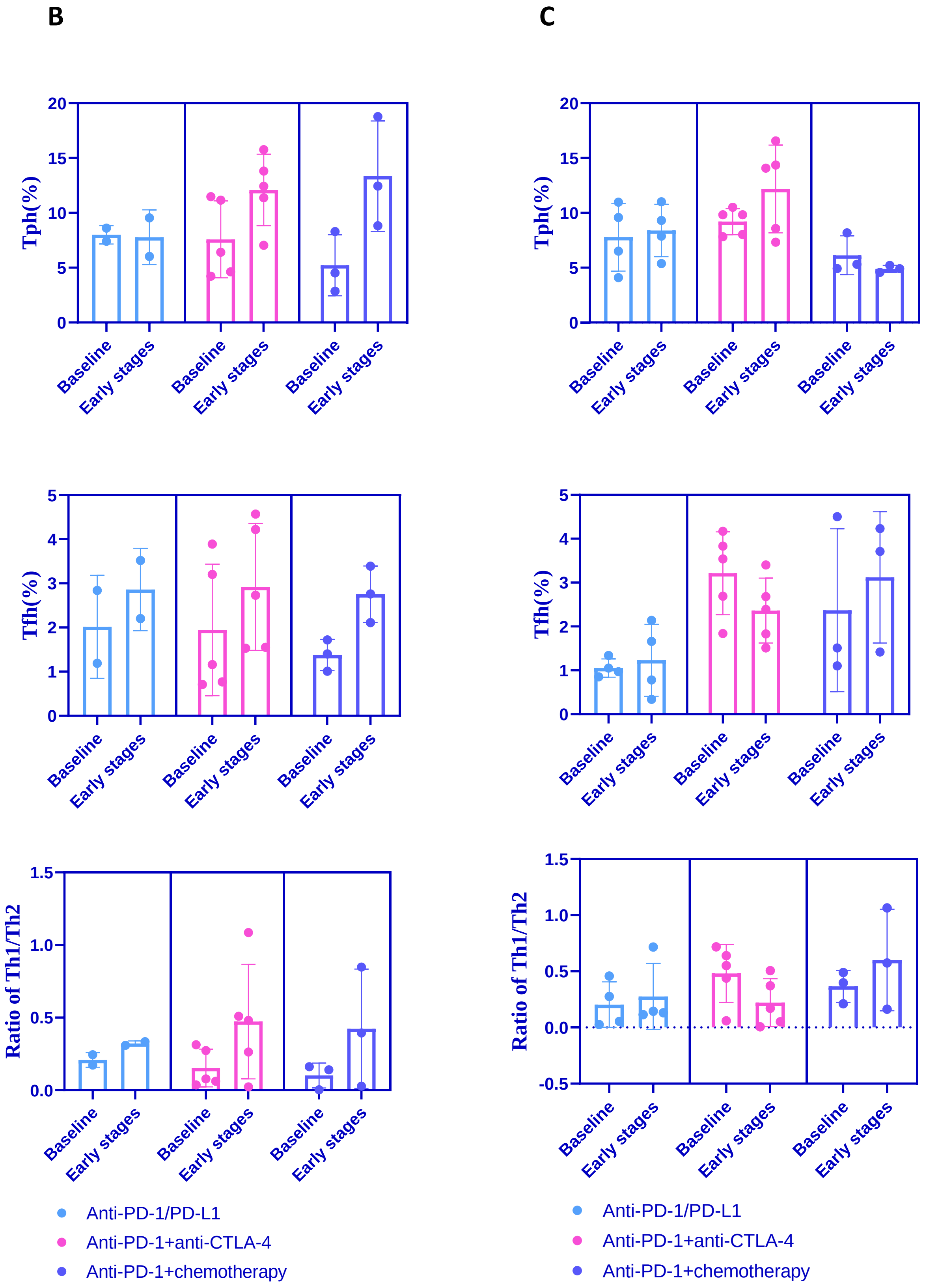
B
C
Tph(%)
Tfh(%)
Ratio of Th1/Th2
Baseline
Early stages
Anti-PD-1/PD-L1
Anti-PD-1+anti-CTLA-4
Anti-PD-1+chemotherapy

Fig 5

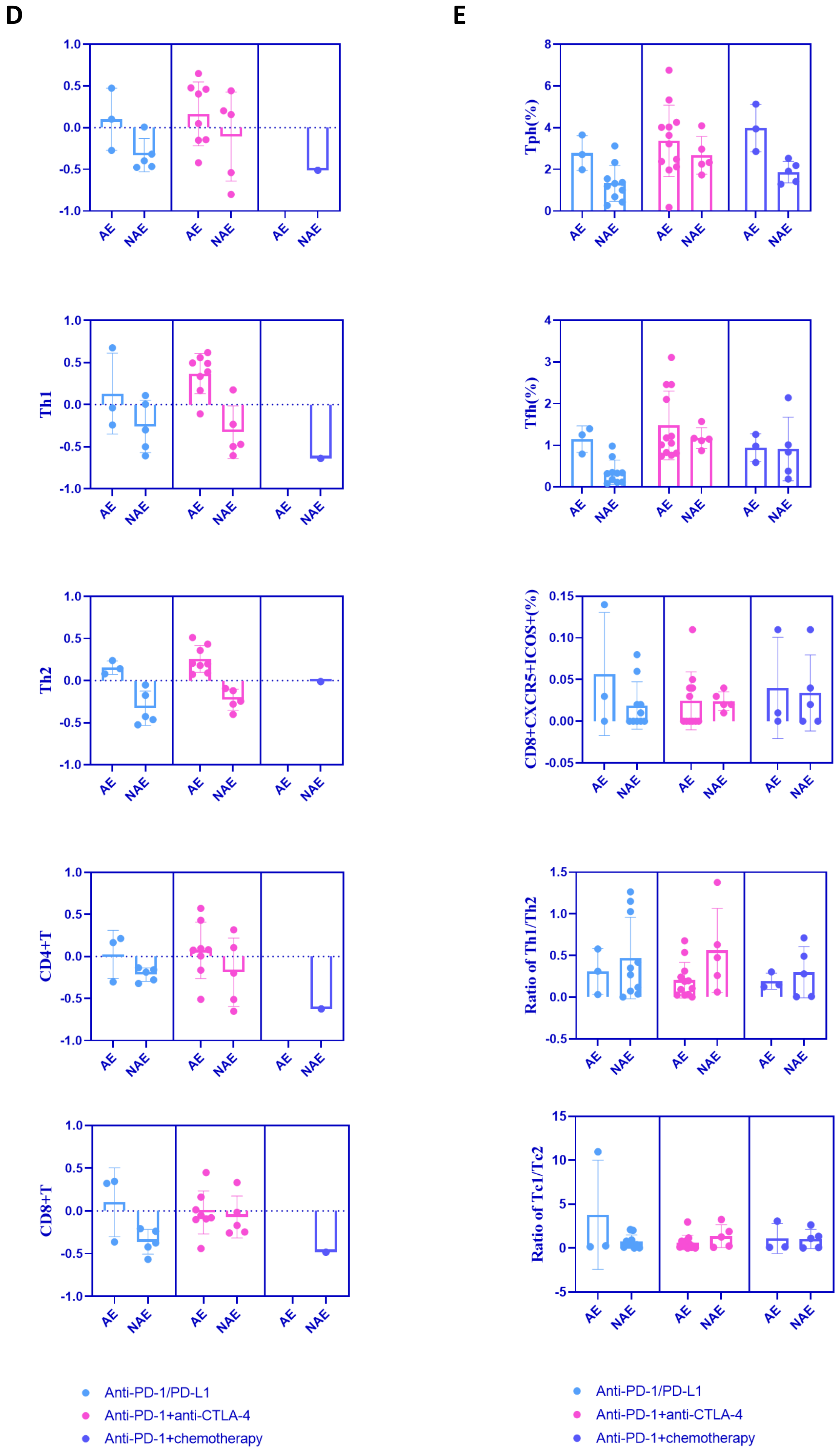

D
E
Th1
Th2
CD4+T
CD8+T
Tph(%)
Tfh(%)
CD8+CXCR5+ICOS+(%)
Ratio of Th1/Th2
Ratio of Tc1/Tc2
AE
NAE
Anti-PD-1/PD-L1
Anti-PD-1+anti-CTLA-4
Anti-PD-1+chemotherapy
